# Assessment of beach litter pollution in Egypt, Tunisia, and Morocco: a study of macro and meso-litter on Mediterranean beaches

**DOI:** 10.1007/s10661-024-13517-x

**Published:** 2025-01-03

**Authors:** Mirco Haseler, Lilia Ben Abdallah, Loubna El Fels, Bouchra El Hayany, Gasser Hassan, Gabriela Escobar-Sánchez, Esther Robbe, Miriam von Thenen, Assala Loukili, Mahmoud Abd El-Raouf, Fadhel Mhiri, Alaa Abdelwahed El-Bary, Gerald Schernewski, Abdallah Nassour

**Affiliations:** 1 Coastal & Marine Management Group, Leibniz-Institute for Baltic Sea Research, Seestrasse 15, 18119 Rostock-Warnemünde, Germany; 2 Tunis International Center for Environmental Technologies (CITET), Tunis, Tunisia; 3 Laboratory of Microbial Biotechnologies, Agrosciences and Environment (BioMAgE) Labeled Research Unit-CNRST N°4, Faculty of Sciences Semlalia, Cadi Ayyad University Marrakech, Marrakech, Morocco; 4 Higher Institute of Nursing Professions and Health Techniques, Essaouira-Marrakech, Morocco; 5 Arab Academy For Science, Technology and Maritime Transport (AASTMT), P.O. Box 1029, Alexandria, Egypt; 6 City for Scientific Research and Technological Applications, New Borg El Arab City, Alexandria, 21934 Egypt; 7 Marine Research Institute, Klaipeda University, Universiteto Ave. 17, 92294 Klaipeda, Lithuania; 8 Waste and Resource Management, Rostock University, Justus-Von-Liebig-Weg 6, 18059 Rostock, Germany

**Keywords:** Monitoring, Sand Rake, Waste mismanagement, Single use plastic, Buried litter, Beach user

## Abstract

We conducted surveys of Mediterranean beaches in Egypt, Morocco, and Tunisia including 37 macro-litter (> 25 mm) and 41 meso-litter (5–25 mm) assessments. Our study identified key litter items and assessed pollution sources on urban, semi-urban, tourist, and semi-rural beaches. Macro-litter concentration averaged 5032 ± 4919 pieces per 100 m or 1.71 ± 2.28 pieces/m^2^, with higher values observed on urban (mean 2.63 pieces/m^2^ ± 3.03) and tourist (mean 1.23 pieces/m^2^ ± 0.91) beaches. Similarly, urban (mean 9.91 pieces/m^2^ ± 12.70) and tourist beaches (mean 5.32 pieces/m^2^ ± 4.48) revealed elevated levels of meso-litter contamination, particularly in the upper third of the beach, which contained the highest quantities both in terms of number (51%) and weight (50%). 55% of the macro-litter and 35% of the meso-litter originated from human shoreline activities and poor waste management. Given the width of some beaches and their high levels of pollution, the standard 100 m macro-litter approach was impractical. To enable cost-effective, long-term monitoring, we adapted it to a faster 10 m transect approach, which provided reliable data on the top 25 litter items, accounting for 82% of beach pollution. Our Sand Rake method effectively quantified pollution on both cleaned and uncleaned beaches, addressing the often neglected meso-litter size fraction. The high pollution levels, top litter items, and identified sources indicate that beach cleaning alone will not solve the pollution problem. Efforts to raise environmental awareness, enhanced waste management, and law enforcement are needed to improve the situation in a sustainable way.

**Supplementary Information:**

The online version contains supplementary material available at 10.1007/s10661-024-13517-x.

## Introduction

Marine litter, defined as “any persistent, manufactured or processed solid material discarded, disposed of or abandoned in the marine and coastal environment” (UNEP 2005) is one of the most significant global marine environmental challenges of our time (Bellou et al., 2021; UNEP, 2009; Urban-Malinga et al., 2020). Marine litter enters the environment in many categories, such as plastic, paper, metal, glass, and others (Ruiz-Orejón et al., 2021) through a variety of sea- and land-based sources and pathways (Veiga et al., 2016). It is found in different size classes such as micro-litter (< 5 mm), meso-litter (5–25 mm), and macro-litter (> 25 mm) (JRC, 2011). Regardless of time and place, plastic represents the vast majority of all marine litter (Reisser et al., 2013; Addamo et al., 2017; European Commission 2018) and it is found all over the world, from urban beaches to the remotest corners of the oceans (Pham et al., 2014).

The effects of marine litter have far-reaching consequences that extend across various sectors, including wildlife, aquaculture, tourism, and shipping (Cesarano et al., 2023; UNEP, 2021; Wagner & Lambert, 2018). In addition, marine litter threatens ecosystem services such as landscape quality, tourism, and recreation (Botero et al., 2017; Maziane et al., 2018; Rangel-Buitrago et al., 2018b). Between 2008 and 2015, marine litter damage in the Asian-Pacific region surged eightfold, costing $10.8 billion in 2015. According to McIlgorm et al. (2022), if plastic production continues as projected, the global costs could reach $229 billion by 2030 and $731 billion by 2050.

The Mediterranean Sea is one of the most polluted areas in the world and faces considerable challenges in relation to marine litter (Galgani et al., 2014; Suaria et al., 2016; UNEP, 2015). Beach tourism is vital to Mediterranean countries, representing around 80% of tourism in coastal regions and serving as a key economic source (Mejjad et al., 2022; UN, 2022). However, the challenges posed by tourism and high coastal population are significant. Mediterranean countries are the primary causes of their own beach pollution (Liubartseva et al., 2018). Approximately 80% of litter originates on land (Serra-Gonçalves et al., 2019), with coastal tourism being a significant contributor to marine and beach litter in the Mediterranean (ARCADIS, 2014). In some tourist areas, more than 75% of annual waste is generated during high season (JRC, 2011). This leads to a substantial increase in beach pollution, up to 4.7 times higher compared to the rest of the year (Grelaud & Ziveri, 2020).

While coastal pollution results from a combination of tourism activities and poor waste management practices (Nachite et al., 2019; Vlachogianni et al., 2018), tourists and beach users also demand clean coastlines. When choosing a local beach, clean sand, and water are the most vital factors (Ariza & Leatherman, 2012; NOAA, 2014), with “clean” often meaning free of litter and algae (Giorgio et al., 2018). Nevertheless, beaches are often polluted, including hazardous litter (sharp-edged and/or toxic), which can make up to 40% of all beach litter (Rangel-Buitrago et al., 2019b). In a survey conducted on beaches in Australia and New Zealand, 21.6% of respondents reported harm caused by beach litter, with 65% of these incidents being wounds (Campbell et al., 2016), and the occurrence of such harmful encounters doubled between 2007 and 2016 (Campbell et al., 2019).

Beach cleaning is often crucial for managing litter levels, and a significant amount of money is spent on regular, professional cleaning efforts. Mouat et al. (2010) calculated the average costs of beach cleaning per km per year in Europe (for 28 different municipalities in Denmark, Ireland, Portugal, Spain, and Sweden) to be on average €7295 (ranging from €171 to €82,101) with the highest costs in tourist areas. For Spanish beaches in Cadiz, the average cost was €50,376 (ranging from €12,050 to €96,150) per km of beach per year (Cruz et al. 2020). Tunisia quadrupled its funding for cleaning 130 km of beaches between 2016 and 2017, reaching a total of around €680,000 or an estimated €5230 per km of beach per year (jeuneafrique, 2017; Tourismeinfo, 2023). Despite significant investment in beach cleaning, pollution remains a persistent problem, which can lead to a decline in visitor numbers and a loss of income and jobs in the tourism sector (UNEP, GRID-Arendal 2016).

Research in Brazil by Krelling et al. (2017) found that over 85% of beach users would avoid heavily polluted beaches (> 15 litter pieces/m^2^), leading to a 39.1% tourism revenue decrease and potential annual losses of US$8.5 million. Coastal areas in Tunisia, Morocco, and Egypt contributed significantly to their GDP in 2018 (5.4%, 7.5%, and 5.1%, respectively) (the Global Economy 2023). However, during high season, these areas face the challenge of managing extensive waste generated by tourists and found on beaches. With tourism expected to increase, this issue becomes even more significant.

To combat marine litter and promote sustainable Mediterranean development, the United Nations Environment Programme (UNEP) launched the Mediterranean Action Plan (MAP). In 2013, MAP introduced the “Regional Plan for Marine Litter Management in the Mediterranean,” providing a comprehensive framework to effectively address pollution (UN, 2013). Here, marine litter characteristics should be evaluated in line with the Marine Strategy Framework Directive (MSFD) of the European Union (EU). In 2016, all Mediterranean countries adopted “The Integrated Monitoring and Assessment Programme of the Mediterranean Sea and Coast” (UNEP/MAP 2016), including indicator 22, which focuses on the assessment of “trends in the amount of litter washed ashore and/or deposited on coastlines (including analysis of its composition, spatial distribution and, where possible, source)” (UNEP, 2017; MSFD TSG ML 2013). Beach litter surveys are vital for monitoring, as coastline litter is a key indicator of marine pollution (JRC, 2011), especially for litter originating from nearby land-based sources (ARCADIS, 2014). Beach surveys are environmentally friendly, cost-effective, and can be carried out by volunteers on a large scale over a long period of time (Haseler et al., 2018, 2020; Schneider et al., 2018).

While EU Mediterranean beaches are monitored seasonally (four times yearly) in regular programs (JRC, 2020; UNEP, 2015), North African monitoring is limited, with few reports on voluntary clean-ups or surveys (Cesarano et al., 2023; UNEP, 2017). Morocco only conducts biannual national macro-litter monitoring on about 20 Mediterranean beaches (MTEDD, 2022). In Egypt, macro-litter monitoring efforts are limited to a regional area east of Alexandria, with no comprehensive nationwide data, while Tunisia has no ongoing national beach litter monitoring.

To monitor beach litter effectively, it is essential to prioritize different beach types. Rural beaches provide insights into background pollution levels and accumulation rates. Monitoring urban and tourist beaches near potential pollution sources allow us to understand land-based contributions and evaluate the effectiveness of litter mitigation efforts. Conducting standing stock surveys at different beaches allows comparison of litter composition, identification of hotspots, awareness raising, is cost-effective and can be used as a starting point for long-term monitoring.

Beach litter monitoring primarily focuses on visible macro-litter (> 25 mm) due to its ease of detection and collection. However, frequent cleaning of urban and tourist beaches, often daily in high season, hampers macro-litter monitoring efforts in areas where it is essential (Haseler et al., 2018). This is especially problematic during the summer when it is impractical to suspend cleaning for extended periods. To address this, it is crucial to find efficient ways to monitor litter on frequently cleaned urban beaches during both high and low seasons, ensuring standardized and comparable results.

One approach is to prioritize meso-litter, a size fraction that has received limited attention and, to the best of our knowledge, is not regularly surveyed in any of the three countries. However, it is an important litter fraction, both numerically abundant and potentially harmful (JRC, 2011). For instance, cigarette butts—one of the most commonly found litter items worldwide (Micevska et al., 2006; UNEP, 2015; Veiga et al., 2016)—are often underestimated in macro-litter surveys (Kataržytė et al., 2020), and their removal remains a significant challenge for both manual and mechanical cleaning (Zielinski et al., 2019). Consequently, meso-litter items, such as cigarette butts and plastic pieces, persist on the beach and accumulate over time (Loizidou et al., 2018), contributing to issues like injuries, scenery degradation, decreased tourism, and potential revenue loss (Araújo & Costa, 2019). Furthermore, these items continue to fragment, resulting in micro-plastics (Okuku et al., 2020).

Studying both macro and meso-litter is crucial to enhance our understanding of beach pollution and litter degradation. This knowledge empowers us to identify significant contributors and prioritize targeted mitigation and avoidance measures, thereby effectively addressing the issue of marine litter.

This research paper analyses beach pollution in Tunisia, Morocco, and Egypt through four survey campaigns. The objectives are as follows:

Analyse beach pollution, including the quantity of litter, top litter items, and litter sources, considering both macro and meso-litter across various beach types, such as urban, tourist, semi-urban, and semi-rural areas.

Investigate the small-scale distribution of litter on beaches and evaluate the feasibility of adopting a modified 100 m method for beach monitoring, especially in heavily polluted or cleaned areas.

Assess the potential of meso-litter monitoring as a replacement or support for macro-litter monitoring, particularly on cleaned beaches.

Serve as a foundational step for long-term monitoring and provide recommendations for its implementation.

## Material and methods

**Fig. 1:**
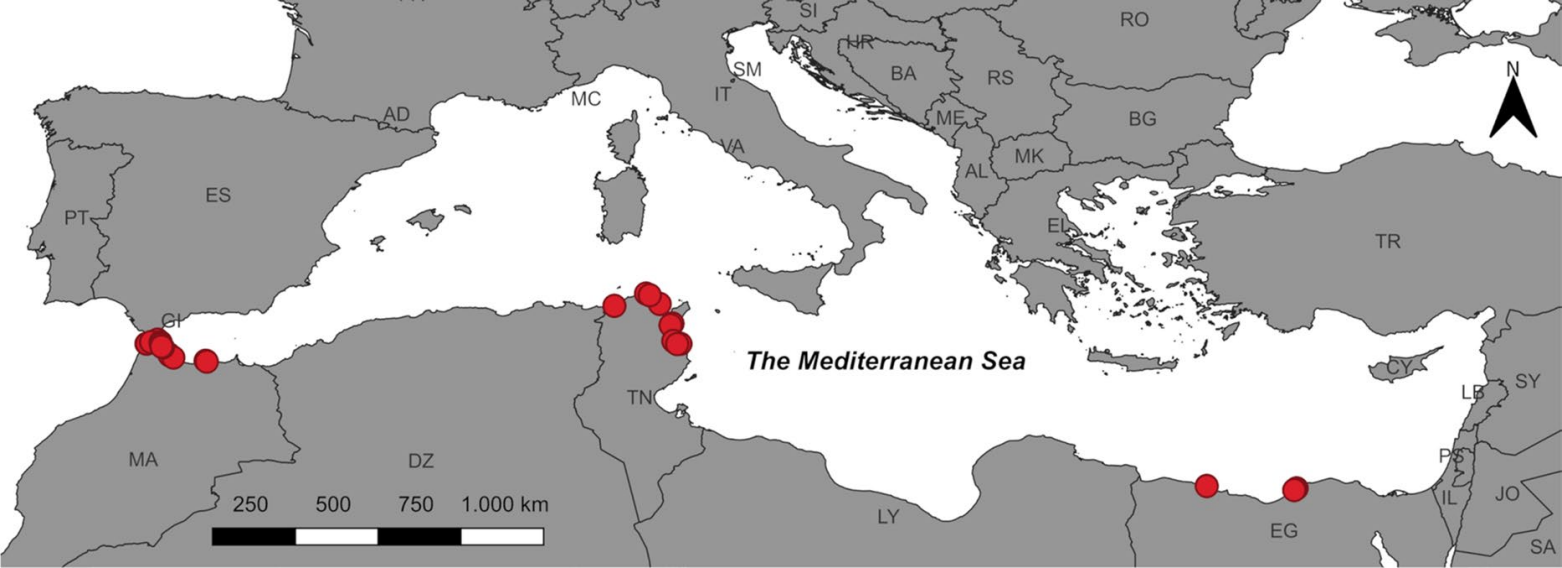
Map of the study area in Morocco (MA), Tunisia (TN), and Egypt (EG). The red dots show the locations of the Macro-litter surveys and the Sand Rake surveys (Map by Eurographics 2020)

Surveys were conducted using two different methods during four campaigns in November 2021 (Tunisia), March 2022 (Egypt), June 2022 (Tunisia), and January 2023 (Morocco), covering different Mediterranean beaches (Fig. 1). The surveys primarily focused on sandy beaches, with some inclusion of those featuring fewer pebbles and rocks. The selection of beaches aimed to provide a partial spatial overview of coastal areas. The beaches surveyed were classified according to their development, using the classification proposed by Semeoshenkova et al. (2017). In addition to the three existing types: urban, semi-urban, and semi-rural—a fourth classification was introduced, termed “tourist.” This addition was motivated by the need to distinguish beaches frequently used by national and international guests from urban beaches primarily used by locals. The Mediterranean Sea is a semi-enclosed system with a small tidal range of around half a meter (Dipper, 2022) and semidiurnal tides (NOAA, 2024). It is only connected to the Atlantic Ocean by the narrow and shallow Strait of Gibraltar (Bergamasco & Malanotte-Rizzoli, 2010).

**Fig. 2:**
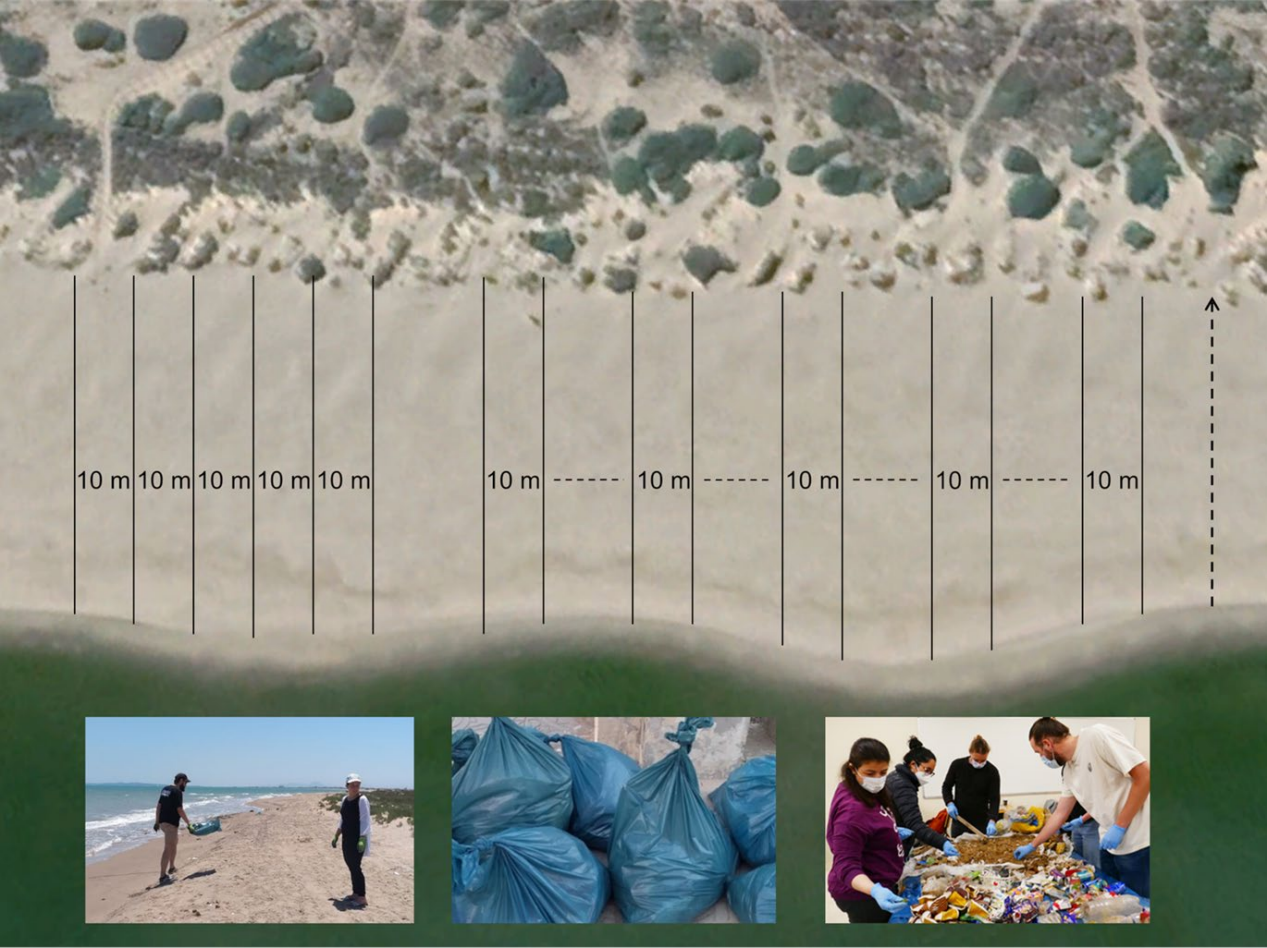
Macro-litter surveys on the beach. On the left side, the 10 m transects were positioned directly next to each other (Tunisia and Egypt). On the right side, larger space of 10–150 m (horizontal dashed line) was used between transects, depending on the length of the beach (Morocco). Vertical dotted black line (on the far right) indicates the Sand Rake method used in direct proximity. The bottom pictures show the survey procedure, which includes systematic litter collection on the beach, placing the litter in plastic bags, and subsequently counting and categorizing the collected litter in the laboratory

The Macro-litter method was used 37 times, in Egypt (8 surveys), Morocco (14 surveys), and Tunisia (15 surveys) (Table 1a supplement material). Surveys were based on the 100 m monitoring method described in UNEP/MAP (2016). However, due to high pollution, resources, and time limitations (aiming for a daily survey), macro-litter was surveyed, counted, and analyzed in 10 m transects (Fig. 2). Beaches were initially categorized by development, followed by surveys conducted by 5 to 10 observers from the waterline to the back of the beach (dunes, cliffs, seawalls, or other structures). The number of 10 m transects surveyed depended on the time required per transect. Due to the limited time for each survey, heavily polluted beaches had fewer transects surveyed. During surveys, litter was systematically collected in labeled plastic bags along each transect. A minimum of two transects were surveyed, with the option to extend up to ten, collecting litter pieces > 2.5 cm. Later, litter from each transect was counted and analyzed. A maximum of 8 h was scheduled for each survey, including litter collection and analysis. Cigarette butts were excluded in the first 2021 Tunisia campaign. In Egypt and Tunisia, transects were placed adjacent to each other, while in Morocco transects were placed 10–150 m apart to allow for a broader comparison of litter distribution.

**Fig. 3:**
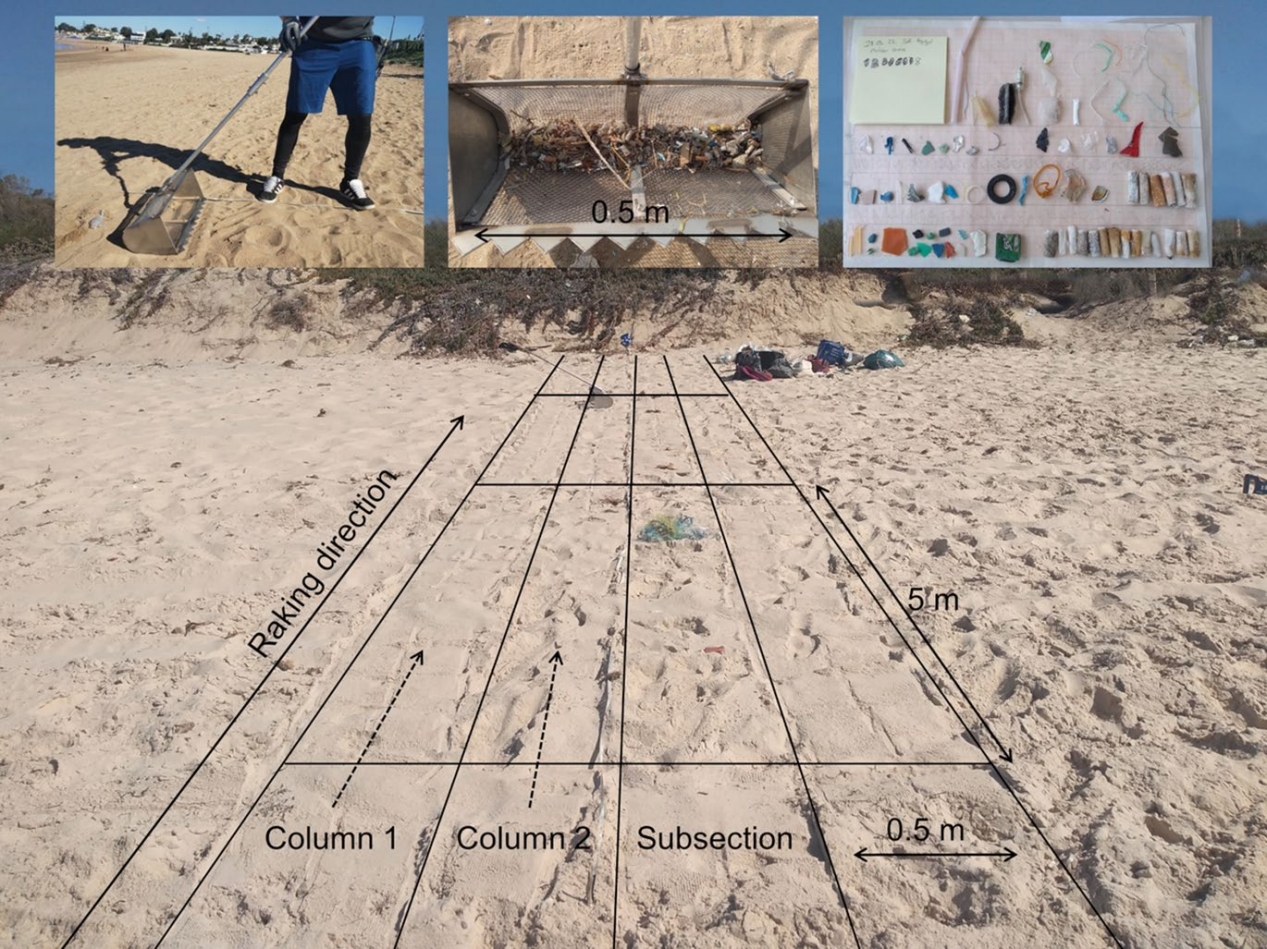
Sand Rake method survey on a sandy beach. Raking direction is from the waterline to the back of the beach (backshore). Operation width of the Sand Rake is 0.5 m; the mesh size used is 5 mm. Each survey is divided in subsections of 0.5 m × 5 m resulting in an area of 2.5 m^2^. Subsections at the back of the beach may be smaller. One survey consists of one or more columns, with different number of subsections. The minimum area per survey is 25 m^2^. The sediment is raked down to a depth of about 5 cm. The upper left picture shows the use of the Sand Rake, while the middle one shows the rake containing litter and organic material, and the right picture shows the analysis of the collected litter, measured and categorized by subsection

The Sand Rake method (Haseler et al., 2018) was used 41 times (Table 2a supplement material) to assess litter on sandy beaches. 19 surveys were conducted in Tunisia, 13 in November 2021, and 6 in June 2022. In Egypt, 10 surveys were conducted in March 2022, while in Morocco, 12 surveys were conducted in January 2023. The surveys focused on the dry backshore of sandy beaches, as the Sand Rake method is unsuitable for wet or coarse sediment. A mesh size of 5 mm was used. The beach width, measured from the waterline to the back of the beach, varied between 10 and 65 m, with a median width of 30 m. The survey process involved raking the beach to a depth of approximately 5 cm in 5 m subsections within columns (Fig. 3). If a column’s area was less than 25 m^2^, additional columns were added to meet the self-imposed 25 m^2^ area requirement. Every column was examined, extending to the back of the beach, regardless of whether the 25 m^2^ target area had already been reached. Per survey, the area ranged from 25 to 50 m^2^, with a median of 30 m^2^. Sand Rake surveys were conducted adjacent to the macro-litter surveys when the sediment was sufficiently fine, with a low number of pebbles, rocks, algae, etc.

Litter analysis for both methods was conducted using the Joint list of litter (j-list) (Fleet et al., 2021) and the allocated online photo catalogue of the j-list (EU, 2021). The highest level of detail was used for litter identification. Nine new litter items were added, as these items were frequently found on the beaches. New litter items in the artificial polymer category were medical masks; broom bristles; plastic strings from carpets; clothes pegs; sand bags and pieces; shisha tips and related; and paint particles. In the category paper/cardboard paper, tissues were added. In the category processed/worked wood, cotton candy sticks were added. All collected litter was categorized to determine if it belonged to the group of single-use plastics (SUP), as defined by the EU Single-Use Plastic Directive (EU, 2019/904) (EU, 2019).

For the macro-litter method, the pollution was measured in litter pieces per 100 m and pieces/m^2^. If a macro-litter survey covered less than 100 m, the average transects pollution was extrapolated to represent a 100 m stretch, following the UNEP/MAP (2016) recommendation for heavily polluted beaches. For the Sand Rake method, the pollution was calculated in pieces/m^2^.

The potential sources of litter were determined using the “matrix scoring technique” system E, as recommended in Veiga et al. (2016), following the method by Tudor and Williams (2004). The matrix is based on likelihoods, which consider the possibility that each litter item may originate from more than one source. The potential sources of litter were classified in eight major categories according to Vlachogianni et al. (2018) and Vlachogianni (2019) and are shoreline, including poor waste management practices, tourism, and recreational activities; fisheries and aquaculture; shipping; fly-tipping; sanitary and sewage related; medical related; agriculture; and non-sourced. A score indicating the potential source was assigned to each litter item within the top 25 of each survey campaign. Local experts familiar with the surroundings and beach activities provided assistance in this process. Litter items with a share of less than 1% of the total findings per country were not allocated to a source. The different likelihood scores are as follows: not considered/impossible (0), very unlikely (0.25), unlikely (1), possible (2), likely (4), and very likely (16). With this percentage allocation, each litter item is assigned to several possible sources on a percentage basis.

The Clean Coast Index (CCI) evaluates coastal cleanliness. To do this, macro-litter is counted per m^2^ of the transect area (length x beach width), using a coefficient (K = 20) for simplification (Alkalay et al., 2007). For the macro-litter surveys in our study, the average pollution of all surveyed transects per beach survey was taken as the basis for the pollution in litter pieces/m^2^. The CCI scale grades the pollution from 0 to 2 very clean beaches, 2–5 clean, 5–10 moderately clean, 10–20 dirty, and > 20 extremely dirty.

$$CCI= \frac{\text{Total of all litter pieces of all transects}}{\text{Total area of all transects}} \times \text{ K}$$

The Hazardous Items Index (HII) was used to assess the risk posed by hazardous litter items on the beach. Hazardous litter includes sharp objects (e.g., metal, glass, bricks) and toxic items (e.g., cigarette butts, medical and sanitary waste). The HII evaluates beach quality based on the amount of hazardous litter present, classifying beaches into five types, with the HHI (number of hazardous pieces per square meter). HHI is the number of hazardous litter pieces/m^2^, taking into account the existing relation between hazardous litter pieces and the log 10 of the total number of all litter pieces found per survey (area in m^2^). The five types are as follows: I (No hazardous litter is seen; HHI 0); II (Some hazardous litter is seen over a large area; HHI 0.1–1); III (A considerable amount of hazardous litter is seen; HHI 1.1–4); IV (A lot of hazardous litter is on the beach; HHI 4.1–8); V (Most of the area is covered by hazardous litter; HHI + 8) (Rangel-Buitrago et al., 2019b). In this study, the HII was calculated for each macro-litter survey.

$$HII= \frac{\frac{\sum Hazardous litter pieces }{log10 \sum Total litter pieces}}{\text{ Area }} \times 20$$

To evaluate the small-scale variability of macro-litter within surveys involving multiple 10 m transects, the coefficient of variation (CV) for pollution levels (per m^2^) across two, three, four, and five directly adjacent transects, as well as the separated transects (Morocco) was calculated. This analysis aimed to assess the variability of pollution in close proximity within each beach survey.

$$CV= \left(\frac{\upsigma }{\upmu }\right)\times 100$$

The frequency of macro-litter items was assessed in each of the 22 beach surveys where five or more 10 m transects were surveyed. The analysis focused on the aggregated top 25 litter items from all 10 m transects per beach survey. This was followed by an assessment of how many transects (1–5) contained these top 25 litter items. If more than five transects were surveyed, a random selection of five transects was analyzed. The objective of this was to determine the number of transects that contained the top 25 litter items, gain insight into their distribution across the surveyed area, and establish the minimum number of transects needed to find all these items.

Litter weight was measured by weighing the litter collected using the Sand Rake method on an electronic scale model: G&G PLC-6000. Prior to weighing, all litter was air dried at room temperature for several days, and sand was removed with a small brush if necessary.

Polymer analysis was carried out using near-infrared spectroscopy (NIR) on 10% (randomly selected) of the non-identifiable plastic pieces collected by the Sand Rake method. For this, the Microphazir PC was used, offering polymer identification and accuracy (in %) within 5 s with a 12 nm (pixels)/8 nm (optical) resolution and 1600–2400 nm spectral range. Only particles with ≥ 95% accuracy were classified, into specific polymer types. Particles with lower accuracy were categorized as unidentified. Black particles were excluded from the analysis as they do not scatter visible light and absorb NIR laser wavelength (Haseler et al., 2020).

## Results

**Fig. 4:**
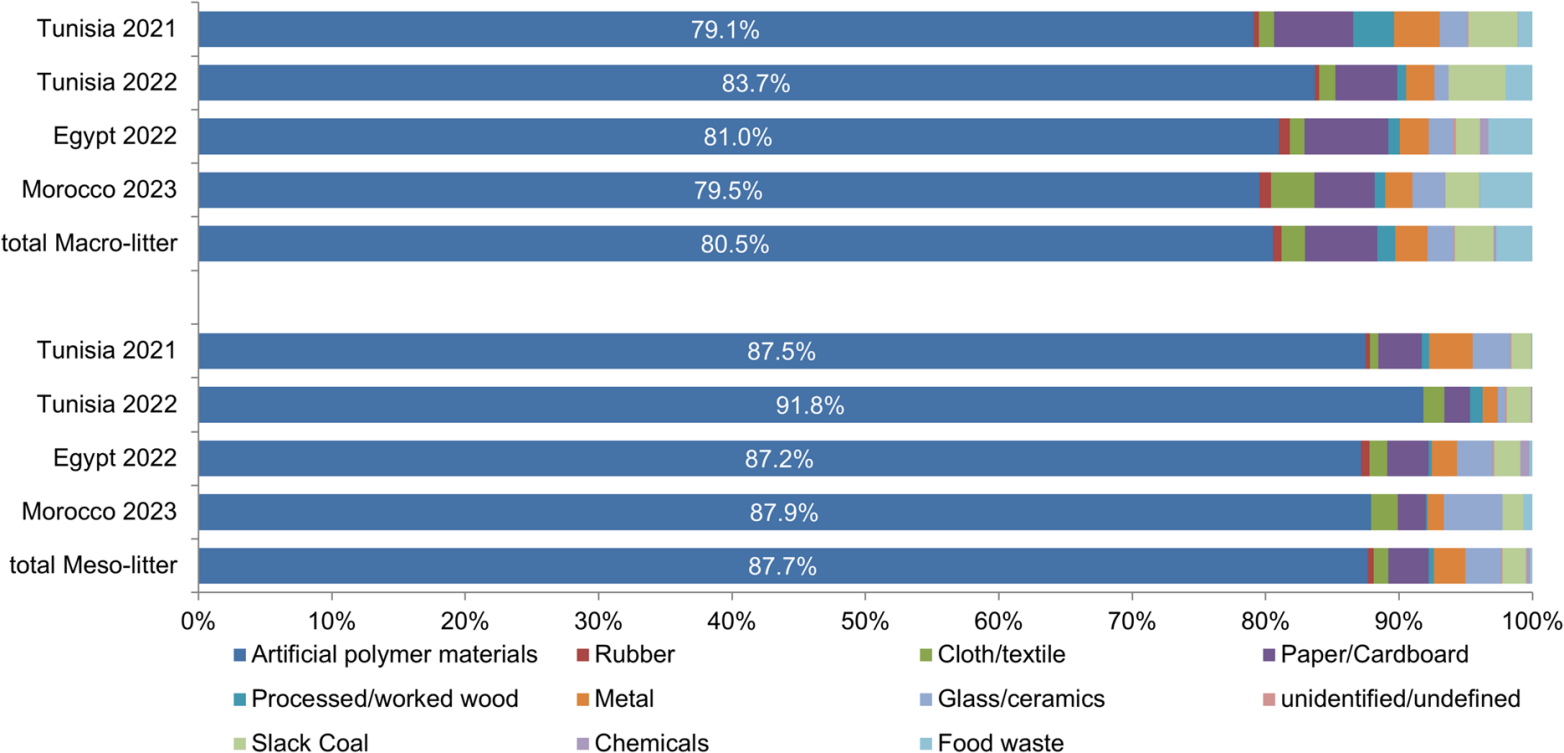
Categories of litter found during the survey campaigns. On top for the macro-litter surveys, and on the bottom for the Sand Rake surveys, with the aggregated results (total)

In 37 macro-litter surveys a total of 71,391 litter pieces, composed of 157 different litter items, were collected on an area of 62,020 m^2^. Both the mean and median number of investigated 10 m transects per survey were five. Across all campaigns, artificial polymers (57,502 pieces) were the dominant litter category, averaging 80.5%, with lesser amounts of other categories (Fig. 4). When extrapolating the findings, litter piece counts ranged from 436 to 24,270 (mean 5032 ± 4919 pieces per 100 m; median 3312). When considering overall beach pollution per m^2^, it ranged from 0.22 to 13.69 litter pieces/m^2^ (mean 1.71 pieces/m^2^ ± 2.28; median 0.99 pieces/m^2^).

In total, 25 litter items were responsible for 81.9% of the pollution. Any other litter item contributed less than 1.0% to the total pollution. The top ten litter items alone accounted for 59.3% of the total litter (Table 1). A total of 32,131 (45.0%) litter pieces were classified as SUP. The most common SUP item was cigarette butts with a share of 20.9% (6709 pieces) of all SUP, followed by crisp packets/sweet wrappers (5212; 16.3%), plastic caps/lids drinks (4993; 15.6%) and 4454 small plastic bags, e.g., freezer bags incl. pieces (13.9%). Regarding the CCI, only one beach, Cap Angela in Tunisia 2022, was classified as clean. Beaches were moderately clean seven times (18.9%), classified as dirty 11 times (29.7%), and as extremely dirty 18 times (48.6%). For details see Table 1a supplement material.

Hazardous litter pieces were found in each survey and ranged from 2.3 up to 49.2% of the total amount of litter per survey (mean 17.5% ± 9.9%; median 16.7%). In total, 12,522 hazardous litter pieces were found (mean 0.28 pieces/m^2^ ± 0.32; median 0.15 pieces/m^2^). The most common ones were cigarette butts (6709 pieces; 53.6%); Glass bottle pieces (734 pieces; 5.9%) and plastic cutlery (716 pieces; 5.7%). In 20 surveys some hazardous litter was found (0.1–1; Type II); in 12 surveys a considerable amount of hazardous litter was found (1.1–4: Type III); in five surveys a lot of hazardous litter was found (4.1–8; Type IV); and in none of the surveys, most of the area was covered by hazardous litter (+ 8; Type V). No significant difference in hazardous pollution was observed along the different beach types. For details see Table 1a supplement material.

### Main pollution of each individual survey campaign

In Tunisia’s November 2021 surveys (8 in total), 17,700 litter pieces were found on 14,160 m^2^ (mean 2.73 pieces/m^2^ ± 4.19; median 1.20 pieces/m^2^). Pollution ranged from 1022 (0.41 pieces/m^2^) to 12,325 (13.69 pieces/m^2^) per 100 m (extrapolated results). Due to high pollution, a 100 m stretch was only surveyed once, a 50 m stretch was surveyed six times, and a 20 m stretch was surveyed once. 8234 litter items (46.5%) were classified as SUP, with five of the top ten litter items being SUP (Table 1).

**Table 1:** Top ten litter items found in the macro-litter surveys in total numbers, percentage and cumulative percentage. Note that no cigarette butts were surveyed in Tunisia 2021. Single-use plastic items were labelled with SUP

	Tunisia 21	Tunisia 22	Egypt 22	Morocco 23	total
1	Plastic pieces 2.5 cm > < 50 cm	Plastic pieces 2.5 cm > < 50 cm	Cigarette butts and filters (SUP)	Plastic pieces 2.5 cm > < 50 cm	Plastic pieces 2.5 cm > < 50 cm
	2546/14.4%	2023/17.3%	3662/16.8%	2720/13.4%	10055/14.1%
2	Plastic caps/lids drinks (SUP)	Plastic caps/lids drinks (SUP)	Plastic pieces 2.5 cm > < 50 cm	Cigarette butts and filters (SUP)	Cigarette butts and filters (SUP)
	1802/10.2%/ 24.6%	1071/9.2%/26.5%	2766/12.7%/29.5%	2350/11.6%/25.0%	6709/9.4%/23.5%
3	Crisp packets/sweet wrappers (SUP)	Polystyrene pieces 2.5 cm > < 50 cm	Small plastic bags, incl. pieces (SUP)	Crisp packets/sweet wrappers (SUP)	Crisp packets/sweet wrappers (SUP)
	1674/9.5%/34.0%	791/6.8%/33.3%	1873/8.6%/38.1%	1299/6.4%/31.5%	5212/7.3%/30.8%
4	Shopping Bags incl. pieces (SUP)	Small plastic bags, incl. pieces (SUP)	Crisp packets/sweet wrappers (SUP)	Plastic caps/lids drinks (SUP)	Plastic caps/lids drinks (SUP)
	1311/7.4%/41.4%	762/6.5%/39.8%	1487/6.8%/45.0%	1158/5.7%/37.2%	4993/7.0%/37.8%
5	Small plastic bags, incl. pieces (SUP)	Crisp packets/sweet wrappers (SUP)	Industrial packaging, plastic sheeting	Polystyrene pieces 2.5 cm > < 50 cm	Small plastic bags, incl. pieces (SUP)
	1022/5.8%/47.2%	752/6.4%/46.2%	1282/5.9%/50.9%	853/4.2%/41.4%	4454/6.2%/44.0%
6	Slack/Coal	Cigarette butts and filters (SUP)	Plastic caps/lids drinks (SUP)	Foam sponge / foamed plastic items	Polystyrene pieces 2.5 cm > < 50 cm
	657/3.7%/50.9%	697/6.0%/52.2%	962/4.4%/55.3%	801/4.0%/45.3%	2502/3.5%/47.5%
7	Polystyrene pieces 2.5 cm > < 50 cm	Slack/Coal	Straws and stirrers (SUP)	Small plastic bags, incl. pieces (SUP)	String and cord (diameter less than 1 cm)
	629/3.6%/54.2%	499/4.3%/56.5%	816/3.7%/59.0%	797/3.9%/49.3%	2138/3.0%/50.5%
8	Cotton bud sticks (SUP)	String and cord (diameter less than 1 cm)	Food contaimers incl. fast food containers (SUP)	Food waste (galley waste)	Industrial packaging, plastic sheeting
	616/3.5%/57.9%	490/4.2%/60.7%	758/3.5%/62.5%	796/3.9%/53.3%	2108/3.0%/53.5%
9	Paper fragments	Industrial packaging, plastic sheeting	Food waste (galley waste)	Carpet plastic string	Slack/Coal
	611/3.5%/61.4%	416/3.6%/64.2%	720/3.3%/65.8%	748/3.7%/56.9%	2067/2.9%/56.4%
10	String and cord (diameter less than 1 cm)	Straws and stirrers (SUP)	String and cord (diameter less than 1 cm)	Lolly sticks (SUP)	Food waste (galley waste)
	505/2.9%/64.3%	344/2.9%/67.2%	653/3.0%/68.8%	740/3.7%/60.6%	1942/2.7%/59.3%

During seven surveys in Tunisia in June 2022, a total of 11,681 litter pieces were found on an area of 11,120 m^2^ (mean 1.16 pieces/m^2^ ± 0.92; median 0.99 pieces/m^2^). The pollution ranged from 436 (0.22 pieces/m^2^) to 15,351 (3.20 pieces/m^2^) per 100 m (extrapolated results). The beach surveys included two 100 m stretches, three 50 m stretches, and one 30 m and 20 m stretch each. 4730 litter pieces (40.5%) were classified as SUP. Half of the top ten litter items were SUP (Table 1).

Eight surveys in Egypt in March 2022 revealed a total pollution of 21,762 litter pieces on 9392.5 m^2^ (mean 2.52 pieces/m^2^ ± 1.36; median 2.38 pieces/m^2^). Pollution ranged between 1964 (0.80 pieces/m^2^) and 24,270 (4.95 pieces/m^2^) litter pieces per 100 m (extrapolated results). 11,123 litter pieces (51.1%) were classified as SUP, with six out of the top ten litter items being SUP (Table 1). No 100 m stretches were surveyed due to high levels of pollution. In total, three 50 m stretches, one 40 m stretch, two 30 m stretches, and one 20 m stretch were surveyed. At one beach in Alexandria, a 35 m stretch was sampled as a single transect due to external circumstances.

In Morocco, 14 surveys were conducted in January 2023. A total of 20,248 litter pieces were found on an area of 27,347.5 m^2^ (mean 0.71 pieces/m^2^ ± 0.43; median 0.52 pieces/m^2^). The pollution ranged from 1304 (0.29 pieces/m^2^) to 5558 (3.00 pieces/m^2^) per 100 m (extrapolated results). Altogether 8044 litter pieces (39.7%) were classified as SUP, with five out of the top ten litter items being SUP (Table 1).

### Beach type pollution

**Fig. 5:**
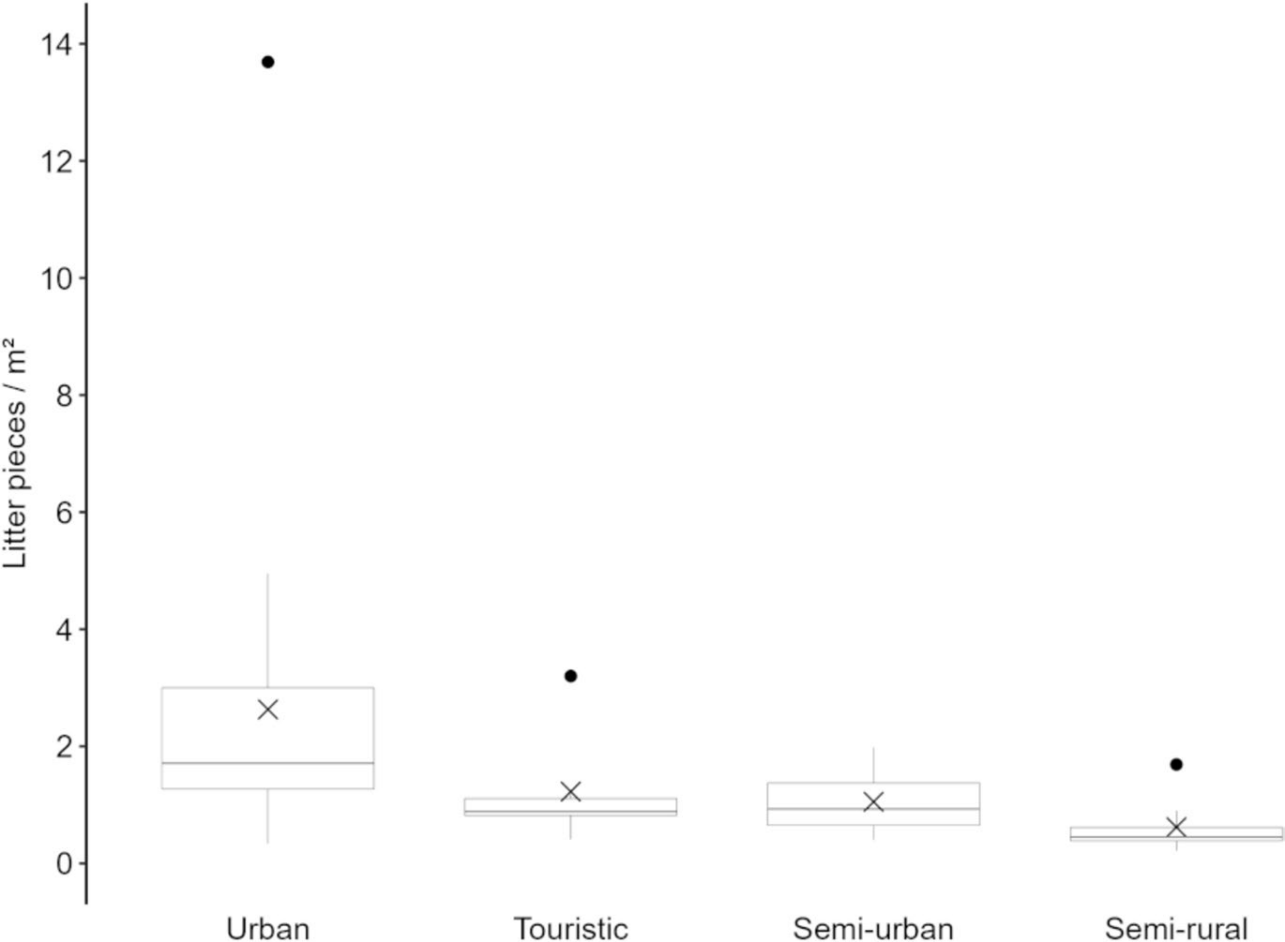
Macro-litter survey campaigns pollution in litter pieces/m^2^ per beach type. Mean value is indicated by cross. Error bars reveal minimum and maximum, dots exhibit outliers

The transnational and cross-campaign analysis of beach types revealed that the eight semi-rural beaches exhibited the lowest pollution levels. The amount of litter ranged from 0.22 to 1.69 litter pieces/m^2^ (mean 0.62 pieces/m^2^ ± 0.45; median 0.46 pieces/m^2^). Per 100 m, the pollution was between 436 and 5558 litter pieces (mean 2196 ± 1500; median 1783). On six semi-urban beaches the litter density ranged from 0.40 to 1.98 litter pieces/m^2^ (mean 1.05 pieces/m^2^ ± 0.54; median 0.93 pieces/m^2^). Per 100 m, the pollution was between 1352 and 6680 litter pieces (mean 3692 ± 1873; median 3784). The six identified tourist beaches had a pollution range of 0.41 to 3.20 litter pieces/m^2^ (mean 1.23 pieces/m^2^ ± 0.91; median 0.88 pieces/m^2^). Per 100 m, the pollution was between 1226 and 15,351 litter pieces (mean 5465 ± 4726; median 3733). On the 17 urban beaches the highest pollution was found, with a range from 0.34 to 13.69 litter pieces/m^2^ (mean 2.63 pieces/m^2^ ± 3.03; median 1.71 pieces/m^2^). Per 100 m, the pollution was between 1022 and 24,270 litter pieces (mean 6687 ± 5939; median 3758) (Fig. 5).

### Litter source allocation

Of 71,391 litter pieces collected throughout the survey campaigns, the top 25 accounted for 58,497 litter pieces (81.9%). These top 25 were used for the source allocation, with four of these top 25 litter items unable to be attributed to specific sources. Those items were plastic pieces 2.5 cm > < 50 cm (10,055; 14.1%); polystyrene pieces 2.5 cm > < 50 cm (2502; 3.5%); foam sponge/foamed plastic items and fragments (1541; 2.2%); and paper fragments (1418; 2.0%). The remaining 42,981 litter pieces were allocated to the litter sources.

**Fig. 6:**
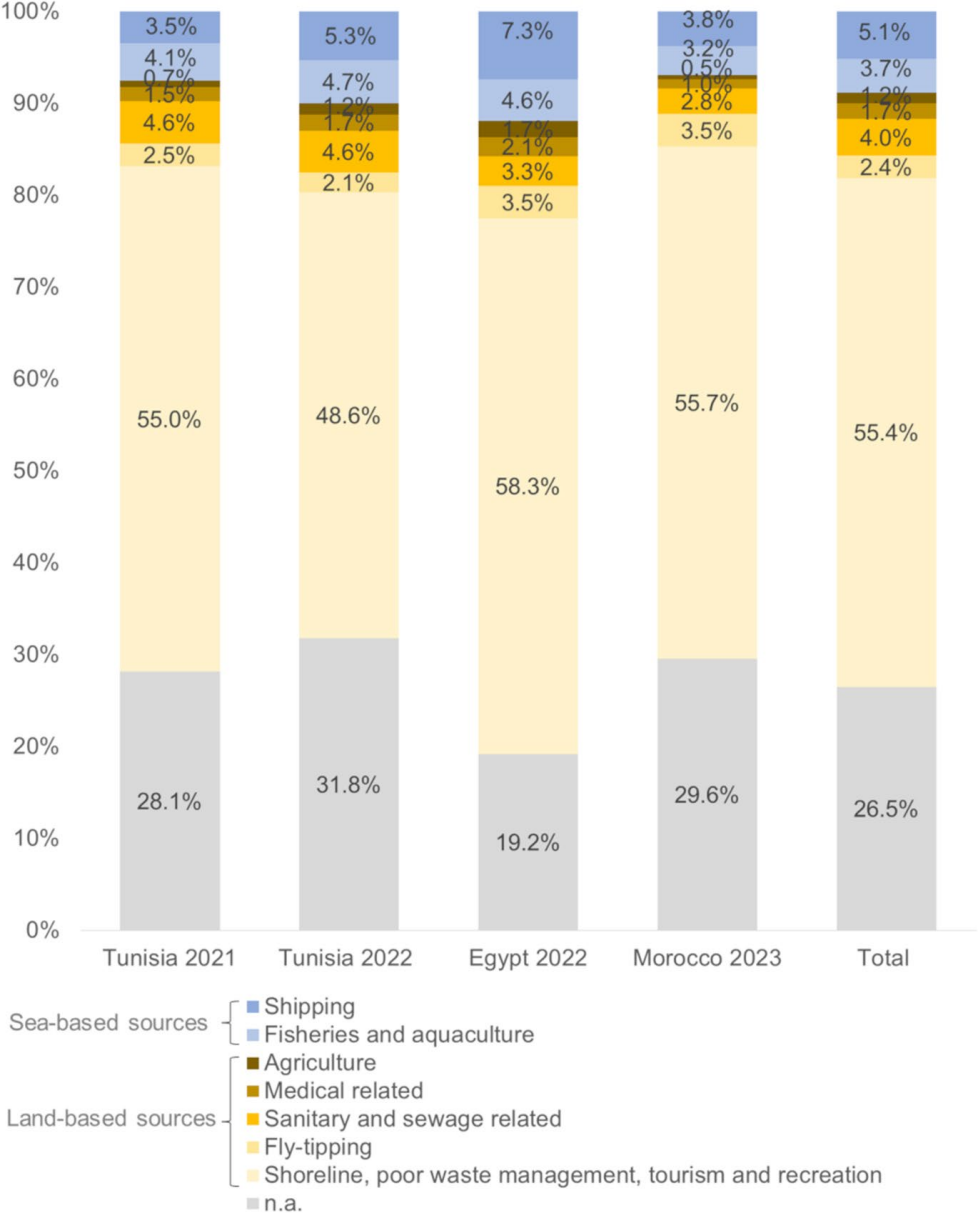
Litter source allocation for the different macro-litter survey campaigns and in total

The percentage of unclassifiable litter varied between the different campaigns, ranging from 19.2 to 31.8% (mean 26.5%). In all survey campaigns, the majority of identifiable litter was attributed to land-based sources and ranged from 58.2 to 68.8% (average 64.6%). In terms of individual sources, most litter was attributed to “shoreline, including poor waste management practices, tourism, and recreational activities” and ranged between 48.6% and 58.3% (average 55.4%). The lower amount of litter originated from the other land-based sources. Sea-based litter attributed with 6.9–11.9% (average 8.8%) to the pollution and is divided between the two sources of “fisheries and aquaculture” with a range from 3.2 to 4.7% (average 3.7%) and “shipping” with an average of 5.1% (range from 3.8 to 7.3%) (Fig. 6). All beach types—urban, tourist, semi-urban, and semi-rural—were equally polluted due to litter from the “shoreline, including poor waste management practices, tourism, and recreational activities” (average 55.4% ± 1.2%).

### Small-scale distribution of litter

**Fig. 7:**
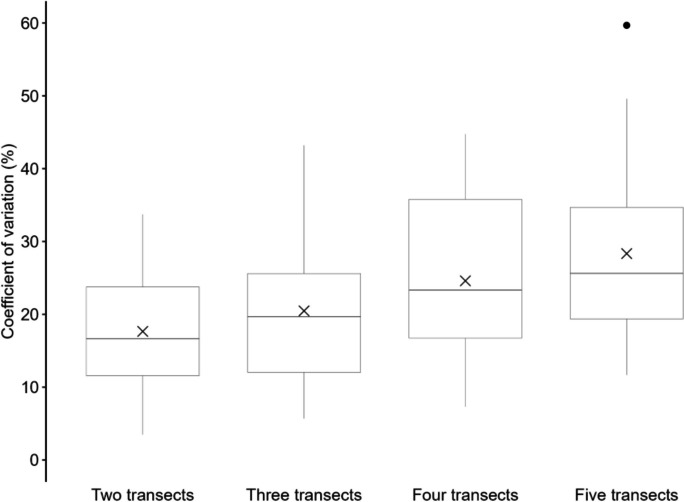
Box-Whisker-Plot of coefficients of variation (CV) [%] for different numbers of adjacent transects (2, 3, 4, and 5). Mean value is indicated by cross. Error bars reveal minimum and maximum, dots exhibit outliers

The pollution measured (litter pieces/m^2^) showed variation across the transects per beach (Fig. 7). Of all the surveys (*n* = 22) analyzed, only four beaches (two adjacent transects) exhibited a CV below 10% (mean 17.7% ± 8.2; median 16.7). When considering surveys (*n* = 19) of three adjacent transects, the CV was twice below 10%, (mean of 21.6% ± 10.8%; median 19.8). For four adjacent transect surveys (*n* = 16), the lowest observed CV value was 7.3%, while no other value fell below 10%. The mean CV was 24.6% ± 11.4; median 23.3%. The highest CV values were recorded in surveys (*n* = 15) where five adjacent transects were analyzed, with the lowest CV of 11.7%. The mean CV was 28.3% ± 13.2; median 25.6%.

Considering the separated transects in Morocco, higher CV values were observed. For surveys (*n* = 4) with transects with a distance ranging from 10 to 50 m, the average CV was 17.7% ± 3.8; median 17.3. For the surveys (*n* = 5) where transects had distances between 50 and 100 m, the CV was higher (mean 24.1% ± 15.1; median 13.6). The highest range of CV was found if the distance between the transects (surveys *n* = 5) was between 100–150 m (mean 23.8% ± 5.4; median 24.8).

### Frequency of the top litter items

**Fig. 8:**
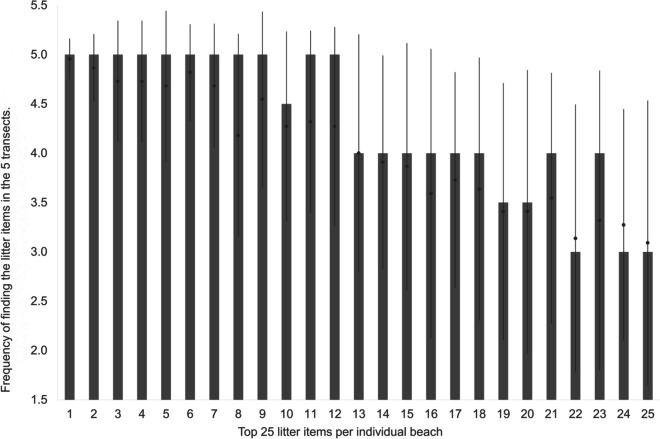
The quantity of transects (*y*-axis) in which each of the top 25 litter items (*x*-axis) was found. The median value is represented by the grey bar with the standard deviation; mean value (black dot)

The analysis, comprising 22 surveys, reveals the presence of the top 25 litter items on the different beaches (Fig. 8). Considering the total values of all top 25 litter items per beach of all surveys, they were found regularly (mean 4.03 ± 1.23; median 5) in the five transects. The 10 most common litter items were found in higher frequency (mean 4.6 ± 0.73; median 5). The other litter items (11–20) were found less frequently (mean 3.81 ± 1.26; median 4). The litter items (21–25) were the least common frequently (mean 3.27 ± 1.37; median 3). On the mean (and median), all of the top 25 litter items can be found within a survey area of 30 m. Considering only the results of Moroccan beaches, where the distance between the 10 m transects per survey ranged from 10 to 150 m, the frequency of litter findings was similar (mean 3.98 ± 1.22; median 4) compared to adjacent transects.

### Sand Rake results

**Fig. 9:**
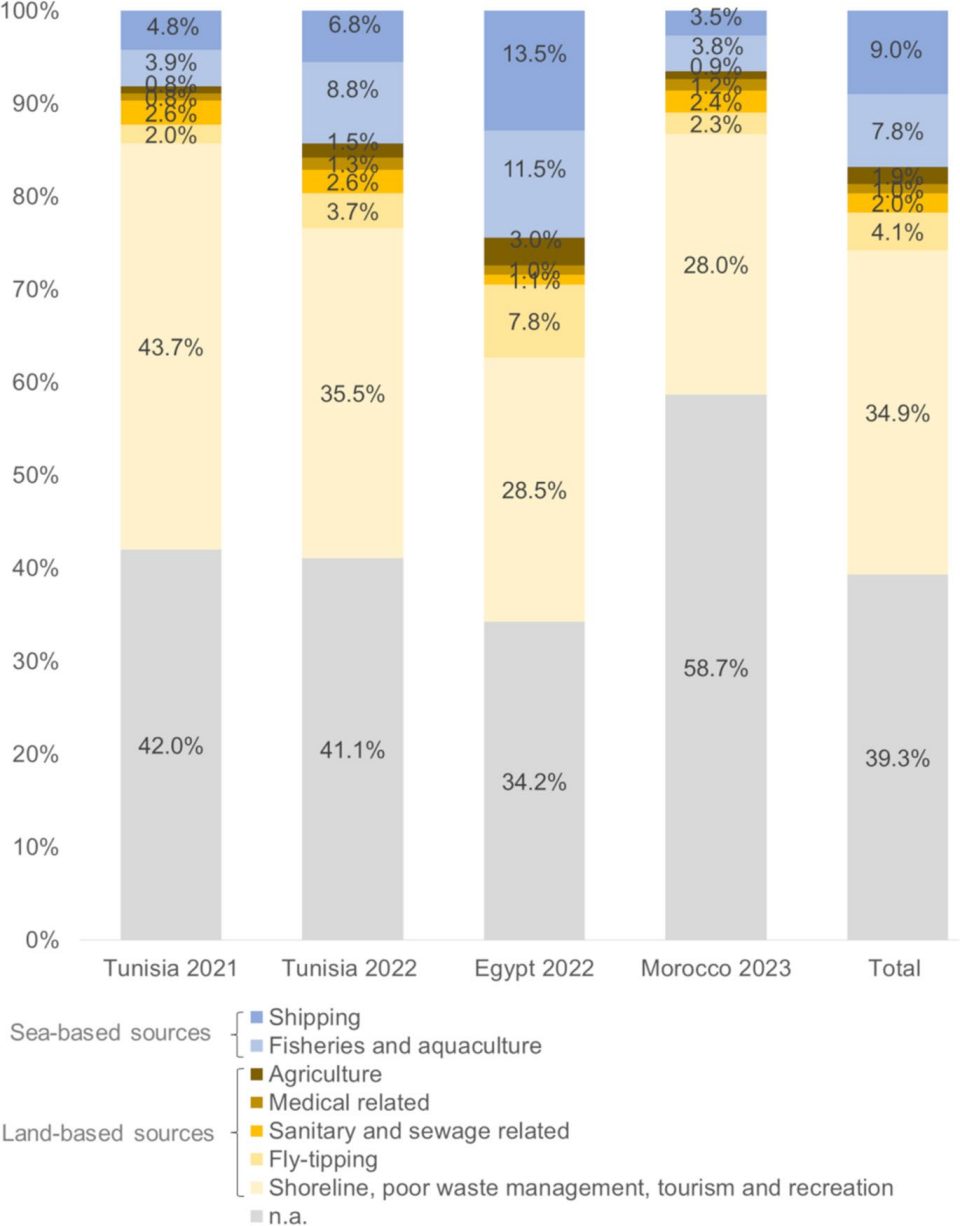
Litter source allocation for the different Sand Rake survey campaigns and in total

The litter collected in all 41 surveys (1287.25 m^2^) of the four campaigns was 12,877 litter pieces (mean 10.00 pieces/m^2^ ± 12.72; median 4.50 pieces/m^2^). The aggregated results per survey showed a maximum of 55.50 litter pieces/m^2^ and a minimum of 0.26 pieces/m^2^. In total, 8376 meso-litter pieces (65.0%) and 4501 macro-litter pieces (35.0%) were collected. Artificial polymers were predominant in all survey campaigns, and had a total share of 11,289 litter pieces (87.2%) (Fig. 4). Other litter categories had a lower percentage. Examining only meso-litter, the pollution ranged between 0.08 and 35.9 litter pieces/m^2^ (mean 6.35 pieces/m^2^ ± 8.93; median 2.38 pieces/m^2^). SUP litter had a share of 32.7% (4204 litter pieces) of the total pollution, with four of the top ten litter items being SUP (Table 2). Most litter could be allocated to land-based sources (Fig. 9).

**Table 2:** Top ten litter items found with the Sand Rake method in total numbers, percentage, and cumulative percentage. Single-use plastic items were labelled with SUP

	Tunisia 21	Tunisia 22	Egypt 22	Morocco 23	all litter
1	Plastic pieces 0.5 - 2.5 cm	Plastic pieces 0.5 - 2.5 cm	Industrial packaging, plastic sheeting	Plastic pieces 0.5 - 2.5 cm	Plastic pieces 0.5 - 2.5 cm
	1285/24.7%	230/26.2%	1447/23.9%	262/34.8%	2871/22.3%
2	Cigarette butts and filters (SUP)	Cigarette butts and filters (SUP)	Plastic pieces 0.5 - 2.5 cm	Plastic pieces 2.5 cm > < 50 cm	Cigarette butts and filters (SUP)
	1048/20.2%/44.9%	98/11.1%/37.3%	1094/18.1%/42.0%	63/8.4%/43.2%	1974/15.3%/37.6%
3	Plastic caps/lids drinks (SUP)	Plastic caps/lids drinks (SUP)	Cigarette butts and filters (SUP)	Cigarette butts and filters (SUP)	Industrial packaging, plastic sheeting
	617/11.9%/56.8%	78/8.9%/46.2%	776/12.8%/54.8%	52/6.9%/50.1%	1727/13.4%/51.0%
4	Plastic pieces 2.5 cm > < 50 cm	Industrial packaging, plastic sheeting	String and cord (diameter less than 1 cm)	Small plastic bags, incl. pieces (SUP)	Plastic caps/lids drinks (SUP)
	268/5.2%/62.0%	70/8.0%/54.2%	433/7.2%/61.9%	35/4.7%/54.8%	878/6.8%/57.9%
5	Industrial packaging, plastic sheeting	String and cord (diameter less than 1 cm)	Polystyrene pieces 0.5 - 2.5 cm	Plastic caps/lids drinks (SUP)	String and cord (diameter less than 1 cm)
	186/3.6%/65.6%	68/7.7%/61.9%	296/4.9%/66.8%	33/4.4%/59.2%	700/5.4%/63.3%
6	String and cord (diameter less than 1 cm)	Plastic pieces 2.5 cm > < 50 cm	Plastic pieces 2.5 cm > < 50 cm	Crisp packets/sweet wrappers (SUP)	Plastic pieces 2.5 cm > < 50 cm
	182/3.5%/69.1%	52/5.9%/67.8%	202/3.3%/70.2%	27/3.6%/62.8%	585/4.5%/67.8%
7	Small plastic bags, incl. pieces (SUP)	Small plastic bags, incl. pieces (SUP)	Crisp packets/sweet wrappers (SUP)	Industrial packaging, plastic sheeting	Polystyrene pieces 0.5 - 2.5 cm
	170/3.3%/72.3%	45/5.1%/72.9%	195/3.2%/73.4%	24/3.2%/66.0%	474/3.7%/71.5%
8	Polystyrene pieces 0.5 - 2.5 cm	Plastic rings from bottle caps/lids (SUP)	Plastic caps/lids drinks (SUP)	Polystyrene pieces 0.5 - 2.5 cm	Small plastic bags, incl. pieces (SUP)
	141/2.7%/75.1%	35/4.0%/76.9%	150/2.5%/75.9%	24/3.2%/69.1%	346/2.7%/74.2%
9	Paper fragments	Crisp packets/sweet wrappers (SUP)	Slack/Coal	Foam sponge / foamed plastic items	Crisp packets/sweet wrappers (SUP)
	126/2.4%/77.5%	24/2.7%/79.6%	119/2.0%/77.8%	21/2.8%/71.9%	322/2.5%/76.7%
10	Plastic rings from bottle caps/lids (SUP)	Slack/Coal	Small plastic bags, incl. pieces (SUP)	Bottles incl. pieces	Slack/Coal
	123/2.4%/79.9%	16/1.8%/81.5%	96/1.6%/79.4%	18/2.4%/74.3%	224/1.7%/78.4%

Tunisia November 2021: During 13 surveys (413 m^2^), a total of 5192 litter pieces were found (mean 12.06 pieces/m^2^ ± 15.49; median 5.83 pieces/m^2^); with a maximum of 55.50 litter pieces/m^2^ and a minimum of 2.37 pieces/m^2^. Artificial polymers accounted for the majority with 4544 litter pieces (87.5%). Considering only meso-litter, the pollution varied between 1.23 and 35.9 pieces/m^2^. 2205 litter pieces (42.5%) of the total pollution were SUP.

Tunisia June 2022: In 6 surveys on an area of 166.75 m^2^, a total of 879 litter pieces were found (mean 5.33 pieces/m^2^ ± 5.82; median 2.07 pieces/m^2^). The maximum pollution found was 17.28 litter pieces/m^2^ and the minimum of 1.04 pieces/m^2^. Artificial polymers were predominant with 807 litter pieces (91.8%). The meso-litter pollution was between 0.08 and 12.12 pieces/m^2^. In total, 879 litter pieces, accounting for 35.5% of the collected litter, were classified as SUP. Five out of the top ten litter items belonged to the category of SUP.

Egypt March 2022: In 10 surveys (345.5 m^2^) a total of 6054 litter pieces were found (mean 17.88 pieces/m^2^ ± 12.91; median 15.41 pieces/m^2^); with a maximum of 47.43 litter pieces/m^2^ and a minimum of 2.34 pieces/m^2^. Artificial polymers dominated the collected litter, accounting for 5277 pieces (87.2%). When considering only meso-litter, the pollution levels varied between 1.69 and 35.15 pieces/m^2^. Out of the total pollution, 1488 litter pieces (24.6%) were SUP.

Morocco 2023: In 12 surveys on 362 m^2^, a total pollution of 752 litter pieces was found (mean 2.02 pieces/m^2^ ± 1.16; median 2.03 pieces/m^2^); with a maximum of 4.53 litter pieces/m^2^ and a minimum of 0.26 pieces/m^2^. Artificial polymers were predominant with 661 litter pieces (87.9%). Considering only meso-litter, the pollution ranged between 0.1 and 4.13 litter pieces/m^2^. Altogether, 199 litter pieces (26.5%) were classified as SUP. 

### Beach type and litter abundance of the Sand Rake surveys

**Fig. 10:**
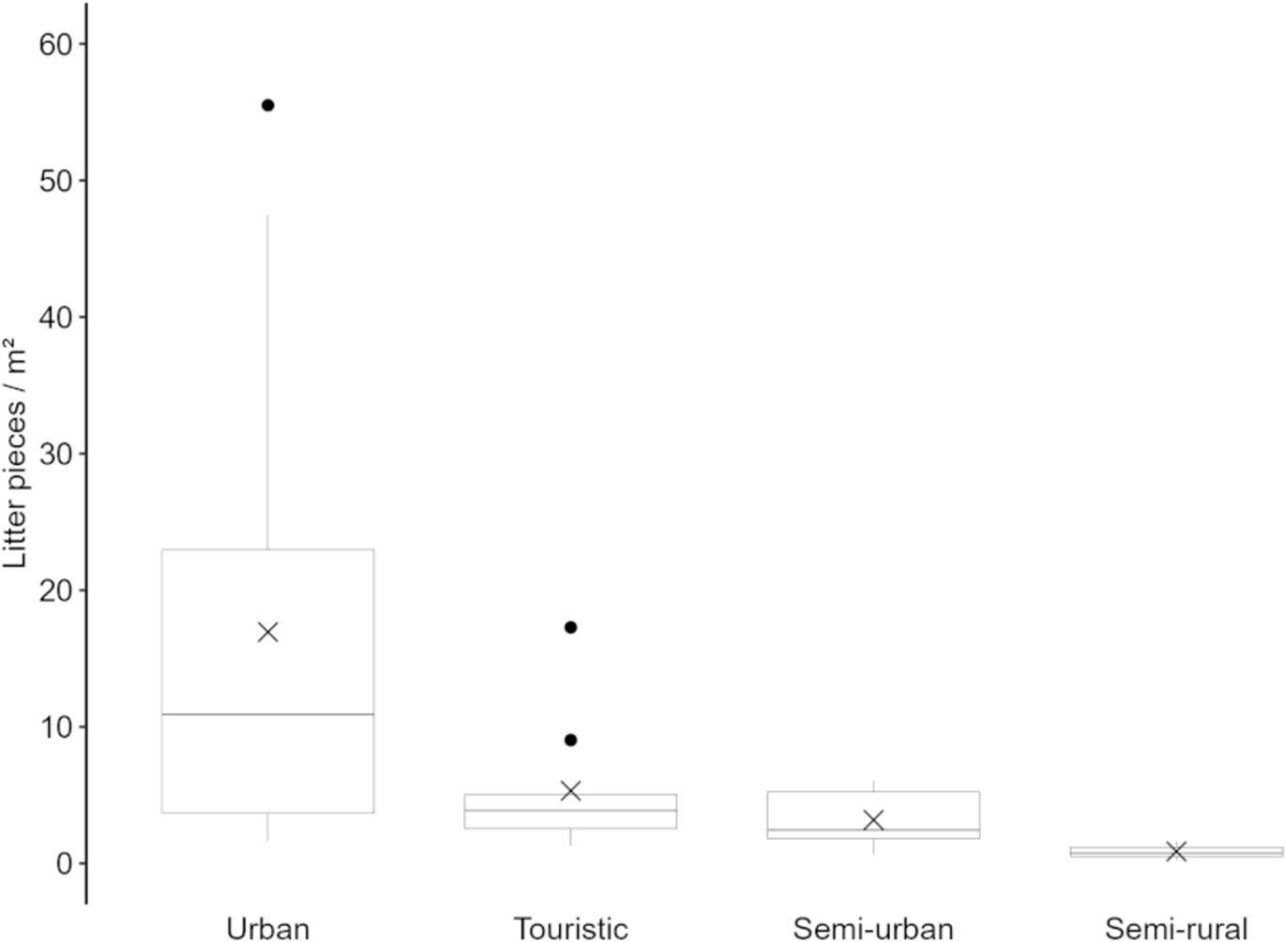
Sand Rake surveys with combined meso- and macro-litter pollution in litter pieces/m^2^ per beach type. Mean value is indicated by cross. Error bars reveal minimum and maximum, dots exhibit outliers

The combined pollution of meso- and macro-litter varied between the different beach types. Semi-rural beaches were found to be the least polluted. The litter density on these beaches ranged from 0.26 to 1.60 litter pieces/m^2^ (mean 0.87 pieces/m^2^ ± 0.55; median 0.76 pieces/m^2^). On semi-urban beaches the litter abundance was slightly higher, ranging from 0.64 to 6.07 litter pieces/m^2^ (mean 3.18 pieces/m^2^ ± 1.95; median 2.44 pieces/m^2^). Tourist beaches exhibited a higher level of pollution between 1.29 and 17.28 litter pieces/m^2^ (mean 5.32 pieces/m^2^ ± 4.48; median 3.87 pieces/m^2^). Among the beach typologies, urban beaches were found to be the most polluted. The litter density on these beaches ranged widely, from 0.26 to 55.50 litter pieces/m^2^ (mean 9.91 pieces/m^2^ ± 12.70; median 4.80 pieces/m^2^) (Fig. 10).

### Litter source allocation—Sand rake

Of the 12,877 litter items collected during the Sand Rake surveys, the top 25 used for source allocation accounted for 11,890 litter items (92.3%). Due to the high amount of fragmented litter found, it was not possible to assign sources to 4678 litter pieces (39.3%) of the top 25 litter items. These were unidentifiable pieces of plastic, polystyrene, and foam in the meso and macro-litter size classes, as well as paper fragments and other textile, glass, and metal fragments. The remaining 7212 litter items could be allocated to the different litter sources. Most of the identifiable litter found originates from land-based sources regardless of the type of beach (Fig. 9) and ranged between 34.5 and 56.7% (average 43.8%).

### Small scale distribution

**Fig. 11:**
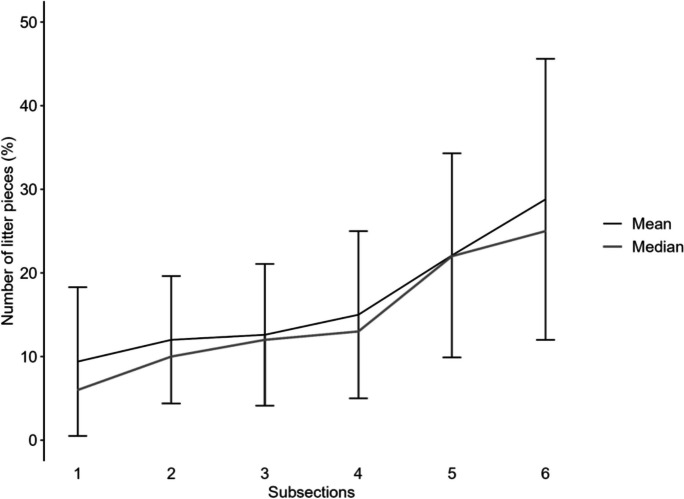
Sand Rake data: Small-scale distribution of litter pieces in % from the lower part of the beach (subsection 1) to the back of the beach (subsection 6), shown for the average beach width of 30 m (6 subsections). Each subsection represents 5 m of the beach. Percentage is shown as mean value with standard deviation and median

Due to variations in beach width, the number of subsections surveyed differed across beaches, ranging from one up to thirteen subsections (mean 6.3; median 6). Examining the small-scale distribution of litter along from the lower part (first subsection) towards the end of the beach (last subsection), it was visible that higher pollution was found on the back of the beach. The results are presented as percentages due to the varying levels of litter pieces/m^2^ on the individual beaches. The combined results indicate that, on average, 50.9% of the litter in terms of numbers was in the upper third of the beach, with 27.6% in the middle section and the remaining 21.5% concentrated in the lower third (Fig. 11). Higher pollution in subsections of the lower or middle part of the beach was mostly due to accumulation zones.

### Weight of litter

**Fig. 12:**
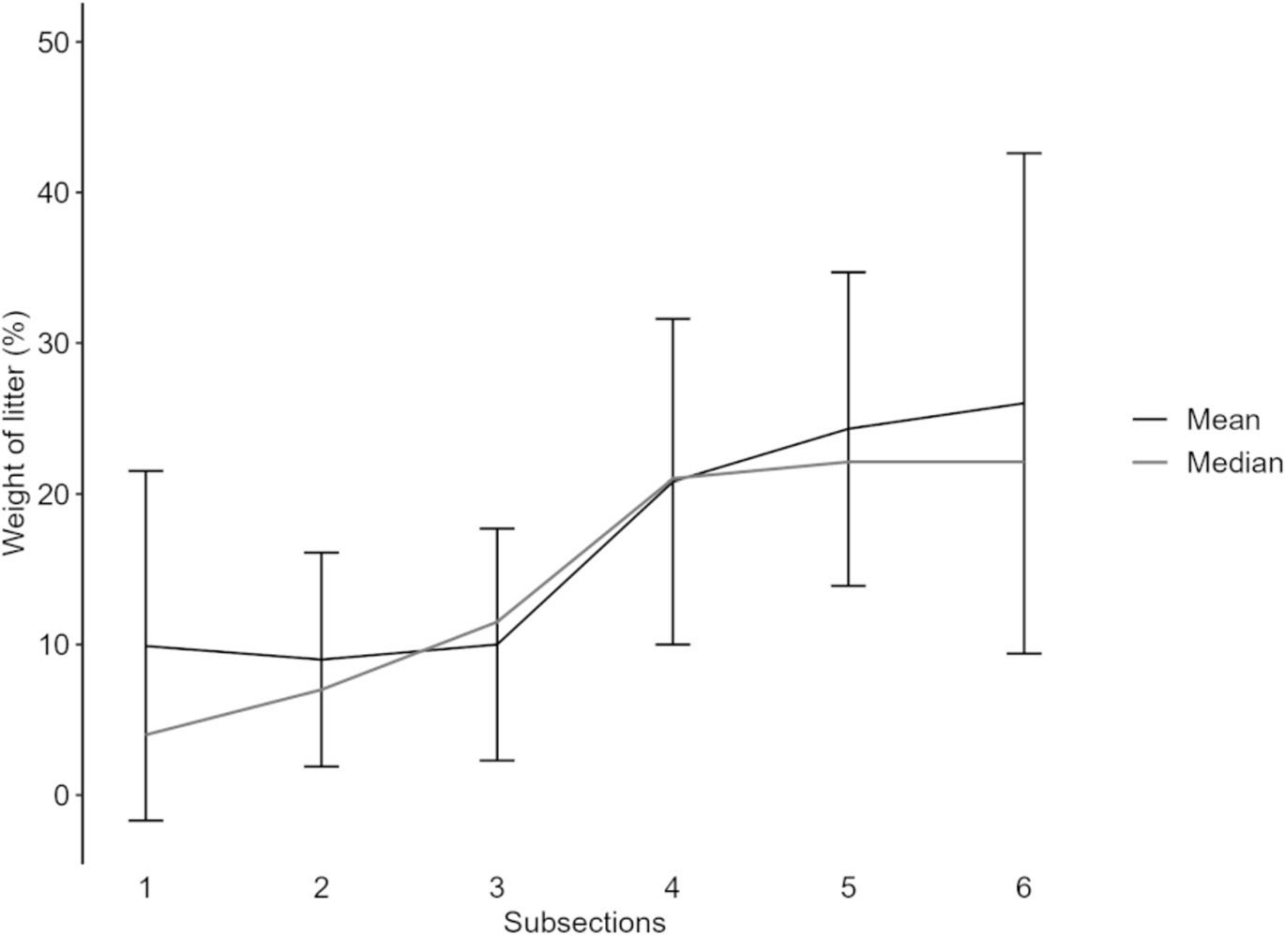
Sand Rake data: Small-scale distribution of litter weight in % from the lower part of the beach (subsection 1) to the back of the beach (subsection 6), shown for the average beach width of 30 m (6 subsections). Each subsection represents 5 m of the beach. Percentage is shown as mean value with standard deviation and median

Litter weight was analyzed for 29 Sand Rake surveys (excluding Morocco). The total average litter weight per m^2^ per beach survey was between 0.25 and 22.66 g/m^2^ (mean 3.63 ± 4.22; median 2.22 g/m^2^). The weight of collected meso-plastic, excluding cigarette butts, ranged from 0.004 to 2.33 g/m^2^ (mean 0.45 g/m^2^ ± 0.54; median 0.22 g/m^2^). For macro-plastic, the weight ranged from 1.4 to 8.04 g/m^2^ (mean 1.40 g/m^2^ ± 1.49; median 1.10 g/m^2^). Calculated for the average six subsections, the highest pollution was on the upper third of the beach. The combined findings reveal that, on average, 50.3% of the litter weight was in the upper third of the beach, 30.8% in the middle section, and the remaining 18.9% in the lower third (Fig. 12).

### Polymer analysis

Using the Sand Rake method, a total of 4045 (31.4%) non-identifiable plastic pieces were found: 2871 plastic pieces (0.5–2.5 cm), 585 plastic pieces (> 2.5 cm), 474 polystyrene pieces (0.5–2.5 cm), and 115 polystyrene pieces (> 2.5 cm). Randomly 10% of the plastic pieces were analyzed with a NIR handheld device and the results were extrapolated to the total amount of non-identifiable plastic pieces. Most of these plastic pieces were composed of polyethylene (PE: 2038 pieces, 50.38%), polypropylene (PP: 1077 pieces, 26.6%), and polystyrene (PS: 589 pieces, 14.6%) and other polymers (90 pieces, 2.2%). Altogether for 251 pieces (6.2%), the polymer type could not be identified.

## Discussion

### Macro-litter pollution

Artificial polymers were the most common litter category found, regardless of country, season, or survey campaign. This reflects the global trend of plastics as the main contributor to marine and beach litter, as shown in other studies (Addamo et al., 2017; Heinrich Böll Stiftung, 2019; Nachite et al., 2019). The top ten litter items in this study’s campaigns are similar to those found in other Mediterranean beach surveys (MTEDD, 2022; Nachite et al., 2019; Vlachogianni et al., 2018). Our findings on SUP accounted for 45% of the pollution, which is comparable to European beaches (43%) where items such as cigarette butts, food containers, plastic bags, and sweet wrappers are also commonly found (European Commission 2018; Vlachogianni et al., 2020).

In this study, we discuss marine litter densities using pieces per m^2^, given that land-based sources dominate. This unit is more appropriate than pieces per 100 m, which is suitable for sea-based sources and associated with floating litter fluxes washed ashore (Vlachogianni et al., 2018).

In a Tunisian study, Rym et al. (2022) investigated three beaches of Monastir (Tunisia) in the four seasons of 2021 and the pollution fluctuated strongly from 0.53 to 8.49 pieces/m^2^ (mean 3.09 pieces/m^2^ ± 2.29; median 2.19 pieces/m^2^). This pollution is comparable to what we found on average in Tunisia 2021 (mean 2.73 pieces/m^2^ ± 4.19; median 1.20 pieces/m^2^) but is nearly 2.5 times higher than our detected pollution in 2022 (mean 1.16 pieces/m^2^ ± 0.92; median 0.99 pieces/m^2^).

Although Morocco has a beach litter monitoring in place for several years, the results are only reported in pieces per 100 m (MTEDD, 2022), making comparisons difficult. However, if we compare our pollution with the national monitoring in autumn 2022, we find that our results are on average four times higher (min 1.2; max 9.3). Between 2015 and 2017, 14 Moroccan Mediterranean beaches were seasonally surveyed and the pollution was 0.054 ± 0.036 litter pieces/m^2^ (Nachite et al., 2019), which is on average 13 times lower than our findings. At a study of five Moroccan Mediterranean beaches the average pollution was 0.20 ± 0.098 pieces/m^2^ (Mghili et al., 2020).

A study conducted in Alexandria, Egypt, in summer 2020 found an average of 7.20 plastic pieces/m^2^ ± 1.03 (Hassan et al., 2022). Based on our findings in Egypt, where 81% of the litter consists of plastic, we estimated a total pollution of 8.57 litter pieces/m^2^ by multiplying this percentage by 1.19. However, our measured pollution (3.03 litter pieces/m^2^) is almost three times lower than the estimated value. The high pollution observed by Hassan et al. (2022) can be partly explained by the lack of beach cleaning in 2020 during the COVID-19 shutdown. Further, a significant amount of litter found by Hassan et al. (2022) was personal protective equipment (PPE). PPE such as gloves and masks (COVID protection) accounted for approximately 40% of the pollution, equivalent to 2.79 ± 0.31 litter pieces/m^2^. In contrast, such PPE items were found only sporadically (0.001/m^2^; 0.09%) in our study.

The percentage of hazardous litter found in our study (mean 17.5%) is lower compared to two studies conducted in Chile, which reported 43% and 28.9% hazardous litter, respectively (Rangel-Buitrago et al., 2019b, 2020) but higher as in a Moroccan study (8.7%) (Bouzekry et al., 2022). Our average pollution level of 0.28 hazardous litter pieces/m^2^ is higher than that of both the Chilean and Moroccan studies, which reported 0.071; 0.15; and 0.22 pieces/m^2^, respectively. The overall high levels of pollution on our beaches are also reflected in the CCI. In our study, 78.4% of the beaches were classified as either dirty or extremely dirty. In contrast, the Chilean studies reported no beaches categorized as extremely dirty, with only 14% and 25% classified as dirty (Rangel-Buitrago et al., 2020) (Rangel-Buitrago et al., 2019b). Similarly, in the Moroccan study, 25% of the beaches were classified as dirty, while the majority were rated as moderate (33.3%) or clean (41.7%) (Bouzekry et al., 2022).

Comparing macro-litter pollution, whether within or across studies, is complex due to factors such as seasonality, beach type, and cleaning practices that significantly influence the observed pollution. For instance, the extended suspension of beach cleaning, particularly in urban and tourist areas like Alexandria, Egypt, during the COVID-19 years likely contributed to elevated pollution levels, providing a snapshot rather than a steady state of pollution. Preliminary results from ongoing macro-litter monitoring in Alexandria (2023) indicate pollution levels at approximately 25–30% of those recorded during our monitoring campaign (personal statement of beach manager). To obtain a more comprehensive understanding of beach pollution, regular monitoring is essential to capture seasonal variations and long-term trends, as discussed further in the recommendations chapter.

### Sand Rake results

The smaller litter fraction was predominantly composed of artificial polymers, which is consistent with findings from other studies (Okuku et al., 2020; Olivelli et al., 2020). The average litter density in this study was 9.86 pieces/m^2^. This is an order of magnitude higher than the 0.91 pieces/m^2^ ± 1.50 reported by Haseler et al. (2020) which used the Sand Rake method on Baltic Sea beaches. There is no other method for meso-litter focusing on the whole backshore of the beach. The following results are therefore only partially comparable with other studies.

Examining meso-litter on 12 Mauritius beaches with 5 mm sieves to a 5 cm depth in five transects perpendicular to the shoreline revealed an average pollution of ~ 4.0 pieces/m^2^ (Mattan-Moorgawa et al., 2021). In a study of 13 Turkish beaches, an average aggregated meso- and macro-plastic concentration was found to be 12.2 ± 3.5 pieces/m^2^ (Gündoğdu & Çevik, 2019). Meanwhile, similar methods in Kenya showed generally higher pollution at tourist beaches (4464 pieces/m^2^ ± 2249.6) compared to remote/rural beaches (328.5 pieces/m^2^ ± 94.0) (Okuku et al., 2020).

In contrast to macro-litter monitoring, it was possible to determine the position of meso-litter along the width of the beach. Our results show a consistent increase in meso-litter pollution from the lower to the upper beach, aligning with findings by Lee et al. (2017) and Okuku et al. (2020) who stated that wind blows litter up the beach. The accumulation of litter in the dunes, which we observed but did not investigate further supports findings of Ryan et al. (2020b) that light litter is carried inland by wind and trapped there. The beaches we studied experience predominantly onshore winds throughout the year (meteoblue, 2024), reinforcing these observations. On the basis of the visual examination and the usual distribution of sediments on beaches (Komar, 1997) we can report that in general, the sediment was finer in the upper part of the beach. As reported by Fulfer and Walsh (2023) litter and sand are transported up the beach and accumulate there, and this finer sediment contains a higher accumulation of litter (Browne et al., 2010; Martins & Sobral, 2011). Furthermore, accumulated litter on the upper beach is less affected by waves, storms, and tides than litter on the surface of lower beach parts (Serra-Gonçalves et al., 2019). Therefore upper beach areas are probably a sink for beach litter.

Olivelli et al. (2020) attribute higher litter densities in the upper beach to Stokes and longshore drift, onshore wind transport, and low departure rates of accumulated litter. Longshore drift moves sand and litter along a beach. It occurs due to the angle of waves hitting the shore, the shape of the coastline, and the direction of the longshore current (NOAA, 2024). Such local beach dynamics play an important role in the distribution of beach litter (Prevenios et al., 2017) and would have provided more information on its (small-scale) distribution, since litter in sediment is most likely to adopt the dynamics of beach sediment transport. But due to the limited time available for each survey, such information could not be determined.

According to Ryan et al. (2020b), only 10.6% of the litter found on the beach is on the surface, with the remaining 89.4% buried in the sediment. Carson et al. (2011) found half of the buried plastic in the top 5 cm of the sediment. To enhance data reliability, we suggest integrating meso-litter surveys with macro-litter surveys. However, it is important to note that most meso-litter studies focus on accumulation zones, which are generally more polluted than other beach areas (Esiukova, 2016; Haseler et al., 2019). Consequently, these results have limited utility in calculating a pollution baseline. Accordingly, we recommend here the Sand Rake method (Haseler et al., 2018; MSFD TSG ML 2023) as it proved useful, particularly on cleaned beaches where macro-litter fractions on the beach surface are often underestimated. Further, it reaches a depth of around 5 cm which helps to find around half of the buried plastic according to Carson et al. (2011). This is important for (long-term) studies and to calculate the total plastic budget of litter on beaches.

Our Sand Rake surveys uncovered that identical litter categories and items were present in smaller sizes fractions and with a higher frequency per m^2^ compared to the macro-litter surveys, this is consistent with other studies (Gyraite et al., 2022; Haseler et al., 2018, 2020). 33% of the litter collected using the Sand Rake method could be classified as SUP, regardless of its small size. However, this is less than in the macro-litter studies because often only plastic fragments were found. But the NIR analysis of such unidentified fragments showed that polyethylene, polypropylene, and polystyrene—polymers often used for SUP—were the dominating polymer types found on the beaches, similar to Haseler et al. (2020), Jeyasanta et al. (2020), and Urban-Malinga et al. (2020). This observation suggests that SUP tends to break down into smaller pieces relatively quickly, potentially explaining the higher percentage (87.7%) of artificial polymers found by the Sand Rake method compared to the Macro-litter method (80.5%).

### Beach type classification and litter sources

Our findings indicate that urban and tourist beaches exhibit higher pollution levels compared to semi-urban and semi-rural beaches, aligning with global trends reported by Poeta et al. (2016), Okuku et al. (2020), Nachite et al. (2019), and MTEDD (2022). 55.4% of the macro-litter could be allocated to tourism, recreation, and poor waste management, regardless of season or country. These results are consistent with other studies around the Mediterranean Sea (Alshawafi et al., 2017; Nachite et al., 2019; Rym et al., 2022; UNEP, 2015; Vlachogianni et al., 2018), where up to 84% of the total pollution originated from recreational and smoking-related activities (Mghili et al., 2020). The litter collected using the Sand Rake method had a higher proportion of unidentified litter (39.3%) compared to the litter collected using the macro-litter method (26.5%). Similar to Meakins et al. (2022), we assume, however, that a significant amount of this unidentified litter, particularly between the high tide line and the back of the beach, originates directly from land-based sources such as beach users. This litter lacked signs of sea origin, such as algae colonization or the distinctive abraded shape resulting from swirling currents or wave action. Furthermore, identifying the source of each litter piece is unnecessary. If the majority of litter is from sources like shoreline, poor waste management, tourism, and recreation, it is logical to conclude that many unidentified pieces share the same origin (Tudor & Williams, 2004). We estimate approximately 70–80% of total litter originates from local or regional land-based sources. This estimate aligns with the proportion of plastic litter from land-based sources in the respective countries (Egypt ~ 84%; Morocco ~ 92%; Tunisia ~ 82%), called the “boomerang effect” in Liubartseva et al. (2018). However, this does not mean that the litter was thrown away or deposited directly where it was found on the beach. Litter can be transported for kilometers along the coast. Sediment drift, driven by currents, waves, and wind, affects how and where litter is shifted. Studies indicate that beaches exposed to stronger winds and currents tend to have higher concentrations of litter compared to more sheltered areas (Camedda et al., 2021). Further, storms cause sediment shifting and as a result, winds can also be the reason for the shifting of beach litter from urban beaches to the less visited beaches (Esiukova, 2016). Wave energy and the beach width are further factors for the state of pollution and determine trends in accumulation and/or backwashing (Bowman et al., 1998). The upper parts of wider beaches with gently slope further seem to be traps for beach litter accumulation as waves can push litter further up the beach (Kataoka et al., 2013; Turrell, 2018). Therefore, in addition to human behavior, beach dynamics need to be further considered when assessing pollution sources of specific beaches, as they are most important for accumulation patterns (Ryan et al., 2009).

Furthermore, the impact of litter of continental origin from watersheds and rivers needs to be considered. For our beaches the Nile River is considered most important. The Nile River, stretching over 6600 km, is the longest river in the world (Soto Chalhoub, 2022), with a watershed covering 2,916,242 km^2^ (Boucher & Bilard, 2020). It is estimated that around 55,000 tonnes of macro-plastic litter enter the Mediterranean Sea annually through the Nile’s watershed, primarily due to rapid urbanization, population growth, littering, inadequate waste management practices, agriculture, and industrial activities. The Nile River itself is considered one of the largest contributors of plastic litter to the Mediterranean, though estimates of its annual macro-plastic input vary significantly. Figures range from 200 to 2000 tonnes (Lebreton et al., 2017), to 6772 tonnes (Liubartseva et al., 2018), and up to 7043 tonnes per year (Schmidt et al., 2017). However, some studies suggest that most plastic litter entering rivers does not reach the oceans but is instead retained within the rivers themselves (van Emmerik et al., 2022) (Schernewski et al., 2024). This could also be the case with the Nile, where the presence of 11 major dams may contribute to the retention of plastic litter along the river’s course (Boucher & Bilard, 2020). As a result, it remains uncertain how much litter entering the Nile far from the Mediterranean reaches the sea, while litter entering closer to the coast has a higher chance of reaching the Mediterranean. In addition to the Nile, numerous of smaller watersheds in our research area contribute plastic litter to the Mediterranean Sea environment, with concentrations ranging from 0.01 to 10,000 tonnes per year (Boucher & Bilard, 2020). Watersheds that extend mainly along the coast have a more direct and immediate impact on plastic pollution along Mediterranean beaches. Lebreton et al. (2017) estimate that at least 11 (temporary) rivers in North Africa contribute to plastic pollution in the Mediterranean, often flowing only during the rainy season or specific periods, with each river releasing between 2 and 200 tonnes of plastic annually. This surely impacts the amount of beach pollution at least seasonally or during high rainfalls and needs to be further focused on in long-term studies including the monitoring of remote beaches that can represent the input of (smaller) watersheds. In general, due to the diversity (size, density, shape) of the litter found, it is often impossible to determine whether or how long a piece of litter has been transported before being collected. But, due to their homogeneity and as one of the most important litter items worldwide, cigarette butts could play an important role as tracers. Loss of mass, degradation, aging, and chemical composition could provide information on how long a cigarette butt has been on the beach (Araújo et al., 2022) and how long or far it has been transported. The density of cellulose acetate, from which cigarette filters are made, is typically around 1.3 g/cm^3^ (Turner & Cundell, 2024) which is similar to beach sand (1.5–1.6 g/cm^3^) (Civil & Structural Engineering 2024). Therefore, cigarette butts could be tracers for the drift of litter along the beach and in the sediment. This could be considered for future beach litter studies.

### Frequency and Small-scale distribution of litter—Macro-litter

The results show that the top 25 litter items are highly variable in terms of the quantity of each item per 10 m transect. This variability increases with more 10 m transects and greater distance between them, making it difficult to determine a representative survey length. This prompts consideration of the effectiveness of a standard 100 m survey versus extrapolating mean values from 10 m transects, especially in terms of time efficiency. A study conducted on a German beach with low pollution levels recommended replicating a similar transect survey approach (Schulz et al., 2021). Considering the high pollution levels of our beaches, we suggest that 3–5 transects per beach would provide reliable information on pollution. The results can be extrapolated to the standard 100 m. Based on our experience, motivating volunteers for regular sampling on heavily polluted beaches has proven difficult. The UNEP reports a similar trend, indicating a 50% decrease in the number of volunteers participating in beach clean-ups from 2002 to 2015 (UNEP, 2015).

### Beach cleaning and steady-state pollution

Research on remote or protected beaches (Merlino et al., 2020; Schulz et al., 2013) suggests that a steady state between litter input and erosion is typically reached within two to three months beaches. However, this may not be the case for our mostly urban and touristic beaches due to several factors, such as the highly developed urban environment, the uncertain impact of the COVID-19 pandemic, the seasonal use of beaches, irregular beach cleaning, unknown and unreliable quality of beach cleaning, poor waste management practices, and other interacting factors within the study areas. Therefore, it is questionable if a steady state between litter inputs from both land and sea-based sources and the outputs resulting from erosion, burial, degradation, and clean-ups will ever achieve.

Beach cleaning is mainly carried out manually by workers during the high season, with an emphasis on macro-litter. Smaller pieces of litter (meso) are often overlooked. Cleaning activities are typically reduced or stopped from September (Nachite et al., 2019), and often only resume at the start of the following high season (personal communication from general beach managers). Pollution continues throughout the year, exposing litter to weathering, sunlight, wind, and mechanical forces that result in fragmentation, transport, size reduction, and burial. As a result, beach sediments contain a significant amount of meso- and micro-litter in addition to macro-litter. Meso-litter in the sediments at the back of the beach remains undetected and untargeted by manual cleaning and accumulates over time (Angelini et al., 2019; Laglbauer et al., 2014; UNEP, 2015). Intense pollution was visible in the dunes and the surrounding hinterland. However, it was not investigated. In general, we assume new beach litter input exceeds litter removal, particularly in the months when no cleaning is carried out.

### Recommendations

To ensure harmonized and comparable results, it is recommended to follow the guidelines for beach macro-litter monitoring (UNEP/MAP 2016; JRC, 2020), where seasonal surveys should be carried out four times per year per beach. Each survey should be carried out in as short a time as possible and not spread over several days (UNEP/MAP 2016). Litter analysis should be carried out by using standardized lists (Fleet et al., 2021) and allocated photo guides (EU, 2021). However, despite having an experienced team, we were only able to complete four out of 37 surveys (incl. litter analysis) over a full distance of 100 m within 8–9 h due to high pollution levels. Therefore, when staffing levels are low and pollution levels are high, it is recommended to shorten the survey to a 30–50 m stretch with a corresponding number of 10 m transects. As our results show a 30–50 m stretch is sufficient to cover the top 25 litter items accounting for more than 82% of the pollution. For affordable and sustainable long-term monitoring, ideally carried out by volunteers, the monitoring process must be time efficient. Otherwise, there is a potential risk of rushed surveys and litter analysis, resulting in poor data quality. The flexibility of the 10 m transect approach allows for adjustments to the number of transects if necessary, and for pollution levels to be extrapolated to the standardized unit of litter pieces per 100 m, while results should also be calculated in litter pieces/m^2^.

It is vital to conduct seasonal surveys including the high season, on all types of beaches. Monitoring urban beaches, which are more impacted by recent land-based litter like “shoreline, including poor waste management practices, tourism, and recreational activities,” enables a rapid evaluation of litter mitigation and avoidance measures (Prevenios et al., 2017). Here, collaboration with professional beach cleaners is essential to prevent survey result bias due to cleaning activities. On urban and tourist beaches with regular cleaning, involving professional cleaners in a study on litter turnover rates could prove beneficial. Collecting and analyzing litter from 10 m transects regularly (for at least 10 days) (Ryan et al., 2020a) provides data on accumulation rate, quantity, weight, and source contributions. Trained staff should conduct the subsequent litter analysis. To gain a comprehensive understanding of pollution drivers, similar surveys should be conducted in parallel at other beaches.

It is further recommended to investigate remote beaches that are difficult for users to access, as described by Rangel-Buitrago et al. (2018b), and that are not subject to any cleaning operations. These remote beaches can be heavily polluted by litter carried by ocean currents, rivers, and smaller watersheds (Rangel-Buitrago et al., 2018a, 2019a, 2019b). Since they are untouched by beach users and unaffected by cleaning efforts, they provide valuable baseline data on pollution levels and sources, offering insights into potential future pollution trends on more accessible urban or tourist beaches if waste management and cleaning practices are not improved. Remote beaches connected to smaller watersheds can reveal the impact of flash floods and heavy rainfall, which often carry large amounts of plastic litter from a variety of continental sources, including agriculture, industry, and urban areas. This litter may have been trapped for years within the watershed before finally entering the Mediterranean Sea environment (Laverre et al., 2023). Flash floods dramatically increase water discharge, leading to sharp surges in macro-plastic flows, with up to 73% of the annual litter from a watershed being released during just a few days of rain events (Laverre et al., 2023).

The consideration of fragmentation and sediment deposition into deeper layers becomes critical in assessing the ultimate presence and mass balance of litter on the beach. Here, Sand Rake results offer insights into the small-scale width-wise distribution of litter, aiding in balancing the plastic budget of beach litter. For a mass balance of beach litter, it is further recommended to analyze the plastic litter weight (MSFD TSG ML 2023). However, this weight analysis can be challenging as the macro-litter method collects a significant amount of litter which is often wet and/or contains sand either in the litter (e.g., in bags or sweet wrappers) or stuck to it. This falsifies the weight results of the litter. Drying and cleaning this sand from the litter is impractical and too time-consuming. In contrast, litter collected using the Sand Rake method is smaller and easier to dry, weigh, and quickly cleaned with a brush. Furthermore, 60.7% of the litter collected using the Sand Rake method was applicable for source allocation and spatial analysis showed that the upper beach is most polluted. Therefore, the method is useful for designing and evaluating targeted litter avoidance and mitigation measures.

Although standing stock surveys can be useful for identifying pollution hotspots and providing an initial overview of litter composition, a comprehensive understanding of litter dynamics requires long-term monitoring over several years. This includes analysis of the influence of external conditions such as wind (direction and speed), tides, sediment drift, and litter deposition in deeper layers. Therefore, initial investigations should include pilot studies to assess the variability within sample data. This should be followed by a power analysis to determine the survey size required to detect a pre-specified change in the amount of beach litter. To improve the accuracy of detecting changes over time, it may be more effective to focus on specific litter items rather than the total amount of litter. For example, concentrating on the most common litter items, cigarette butts, SUP or other land-based litter can provide more precise results. By following these recommendations, more reliable and comprehensive data can be collected, which is essential for the implementation and evaluation of effective measures to reduce and prevent marine litter.

In this context, the use of satellites and drones should be considered to improve beach litter monitoring. Spatially precise monitoring over large areas can be achieved in a harmonized way using satellite remote sensing with multi- and hyperspectral optical sensors (Martínez-Vicente et al., 2019; Paula M. Salgado-Hernanz et al., 2021). Subsequently, drones could be used for more detailed national and regional surveys. As reviewed by Veettil et al. (2022), drones have been used effectively in several beach litter studies and can help identify litter hotspots and litter categories. By comparing the litter identified in situ (from the 10 m transects) with the corresponding drone images, it may be possible to calculate a recovery rate (for different litter items and their quantity) for the drone results. This recovery rate can then be used to project the results to larger areas surveyed by drones alone.

Consideration could also be given to how frequent voluntary beach clean-ups (e.g., Coastal Clean-up Day) could be combined with, for example, drone and macro-litter surveys. In many places around the world, beach clean-ups take place regularly throughout the year, but there is often a lack of systematic data collection during these activities. As a result, important information is lost, that could otherwise be used for ongoing monitoring. A systematic use of data from such clean-ups could improve our understanding of beach pollution trends.

To prevent beach users from migrating to cleaner regions and causing financial losses to the local tourism industry, it is imperative to improve manual beach cleaning and extend it throughout the year. According to Ballance et al. (2000), 85% of beach visitors avoid beaches with more than 2 litter pieces per meter. Our average pollution of macro litter (1.71/m^2^) is close to this value, while the meso-litter exceeds this value by a factor of five. Particularly concerning are the CCI and HII indicators: only one out of 37 beaches is classified as clean (CCI). Hazardous litter was found in every beach survey, and it can be assumed that this type of litter has an even more negative impact on beach users compared to litter that is considered less harmful to humans. The presence of dangerous litter likely heightens health and safety concerns, further diminishing the beach experience. Considering that beach users typically enter from the back, where most litter tends to accumulate, this could further enhance the deterrent effect. Further, the implementation of mechanical beach cleaning should be considered to address the high meso-litter pollution in the upper sediment layers. It should be further evaluated to what extent such mechanical beach cleaning equipment can be used for monitoring purposes.

For almost all Mediterranean countries, beach tourism is of great social and economic importance (Mejjad et al., 2022), yet Mediterranean beaches are highly polluted. Kiessling et al. (2017) found that the extent of the pollution problem is not automatically related to local action; large quantities of litter do not guarantee an adequate reaction from the population or authorities. It is important for the tourism sector, beach managers, and politicians to understand that coastal ecosystems, like beaches are key providers of leisure and recreational ecosystem services (Krelling et al., 2017). However, for these beaches and coastal areas to provide these services effectively, they must be kept clean. People avoid polluted beaches and more time and money is spent visiting clean beaches or pursuing other activities (NOAA, 2014). The experience of a polluted beach reduces the likelihood of return visits and leads to a corresponding loss of revenue (Jarvis et al., 2016). The “Big Fives” are most important to beach visitors: safety, facilities, water quality, no litter, and scenery (Giorgio et al., 2018). The last three can only be achieved by following the principles of sustainable tourism, the 2030 Agenda for Sustainable Development, and the 17 Sustainable Development Goals (SDGs), valuing coastal ecosystems as the very resource on which all beach tourism is based.

Furthermore, it is important to understand, that a sustainable approach and lasting solutions require a shift towards comprehensive marine litter avoidance measures. Considering that nearly half of the litter found is SUP, it is crucial to address the principles of a circular economy and a transition away from the linear use of short-lived plastic. The design of products should aim for durability, recyclability, reuse, and towards the innovation of SUP alternatives. Here, the Extended Producer Responsibility (EPR), where producers are accountable for the entire lifecycle of their products, can play an important role in financing the transition towards a circular economy and promote the production of environmentally friendly products. It is recommended to implement pilot projects for litter monitoring, testing specific measures to reduce litter from tourists, hotels, and other recreational activities, and strengthening the waste management sector.

Although this study has provided information on how to improve beach litter monitoring on heavily littered beaches, it has only focused on the beach surface up to a depth of 5 cm. Future studies should also include the hinterland or dunes, which are often heavily littered but were not part of our study.

## Conclusion

Regional land-based litter sources, specifically poor waste management practices, littering from beach users, tourism, and recreational activities, have been identified as the main contributors to the beach pollution conducted in this study. The consistent dominance of the top 25 litter items, which account for 82% of the pollution in each survey campaign, highlights the need to focus on each of these items with specific avoidance and mitigation measures. The implementation of short- and medium-term mitigation measures such as beach clean-ups, mechanical removal of litter from coastal areas, and the enforcement of policies and regulations to control littering are necessary; especially, as beach tourism plays a significant role of income for Egypt, Morocco, and Tunisia. Clean and litter-free beaches are highly valued by tourists, making their maintenance essential for sustaining tourism revenues and attracting international visitors to the region. Here, a marine litter monitoring program is of great importance to evaluate the effectiveness of mitigation and avoidance measures. It provides essential insights into the progress made in reducing overall marine litter. For a practical and cost-effective approach, we recommend initiating long-term macro and meso-beach litter monitoring using the 10 m transect and the Sand Rake method. These are effective and low-cost methods that can be implemented on a large scale with the help of volunteers.

Supplementary information.

## Supplementary Information

Below is the link to the electronic supplementary material.

Supplementary file1 (PDF 133 KB)

## Data Availability

The datasets generated during and/or analyzed during the current study are available from the corresponding author on reasonable request.
