# Supplementary material for: Assessment of beach litter pollution in Egypt, Tunisia, and Morocco: a study of macro and meso-litter on Mediterranean beaches
Source: Environ Monit Assess. 2025 Jan 3;197(1):123. doi: 10.1007/s10661-024-13517-x (PMC11695667; doi:10.1007/s10661-024-13517-x)
Supplement: Supplementary file 1 — Supplementary file1 (PDF 133 KB) [file 10661_2024_13517_MOESM1_ESM.pdf]

Assessment of Beach Litter Pollution in Egypt, Tunisia, and Morocco: A Study of Macro and Meso-litter on Mediterranean Beaches

Mirco Haseler[1*], Lilia Ben Abdallah[2], Loubna El Fels[3], Bouchra El Hayany[3,4], Gasser Hassan[5,6], Gabriela Escobar-Sánchez[1,7], Esther Robbe[1,7], Miriam von Thenen[1], Assala Loukili[3], Mahmoud Abd El-Raouf [5], Fadhel Mhiri[2], Alaa El-Bary[5], Gerald Schernewski[1,7], Abdallah Nassour[8]

[1]Coastal & Marine Management Group, Leibniz-Institute for Baltic Sea Research, Seestrasse 15, D-18119 Rostock-Warnemünde, Germany
[2] Tunis International Center for Environmental Technologies (CITET), Tunis, Tunisia
[3]Laboratory of Microbial Biotechnologies, Agrosciences and Environment (BioMAgE) Labeled Research Unit-CNRST N°4, Faculty of Sciences Semlalia, Cadi Ayyad University Marrakech, Marrakech, Morocco
[4]Higher Institute of Nursing Professions and Health Techniques, Essaouira-Marrakech, Morocco
[5]Arab Academy For Science and Technology and Maritime Transport (AASTMT) P.O. Box 1029, Alexandria, Egypt
[6]City for Scientific Research and Technological Applications, New Borg El Arab City, Alexandria 21934, Egypt
[7]Marine Research Institute, Klaipeda University, Universiteto Ave. 17, LT-92294 Klaipeda, Lithuania
[8]Waste and Resource Management, Rostock University, Justus-von-Liebig-Weg 6, D-18059 Rostock, Germany

*corresponding author: mirco.haseler@io-warnemuende.de

Table 1a Macro-litter data, of 37 surveys, with beach name, coordinates, country, date of the survey, the distance surveyed, the total amount of litter found, with extrapolated litter results to a 100 m stretch. Further, the area surveyed in m² and extrapolated to the area of 100 m stretch and litter pollution per m². Further, the beach type information, the Clean Coast Index value and numeric index (CCI), and the total amount of hazardous litter, hazardous litter pieces/m², the HII Index, and the HHI Type.

**Beach**	**Coordinates**	**Country**	**Date**	**Distance surveyed [m]**	**Llitter total**	**Litter extrapolated to 100m**	**Area in m²**	**Area extra-polated**	**Litter m²**	**Beach type**	**CCI**	**CCI Index**	**Hazardous litter total**	**Hazardous litter pieces / m²**	**Hazardous Item Index (HII) log 10**	**HHI (Type)**
Hammamet	36°24'7.53"N 10°35'16.97"E	Tunisia	14.11.21	100	1022	1022	1370	1370	0.75	urban	14.92	dirty	24	0.02	0.12	II
Alhambra 1	36°21'7.17"N 10°31'42.86"E	Tunisia	16.11.21	50	3340	6680	2220	4440	1.50	semi-urban	30.09	extremly dirty	212	0.10	0.54	II
Alhambra 2	36°21'19.73"N 10°31'52.08"E	Tunisia	16.11.21	50	613	1226	1490	2980	0.41	touristic	8.23	moderate	64	0.04	0.31	II
Hammam Sousse	35°51'58.34"N 10°36'45.93"E	Tunisia	17.11.21	50	4178	8356	2045	4090	2.04	urban	40.86	extremly dirty	301	0.15	0.81	II
Monastir	35°46'26.91"N 10°50'7.73"E	Tunisia	19.11.21	20	2465	12325	180	900	13.69	urban	273.89	extremly dirty	148	0.82	4.85	IV
Bizerte	37°16'59.46"N 9°52'49.21"E	Tunisia	22.11.21	50	2010	4020	3785	7570	0.53	urban	10.62	dirty	101	0.03	0.16	II
Cap Angela	37°20'1.79"N 9°45'50.97"E	Tunisia	23.11.21	50	1665	3330	1855	3710	0.90	semi-rural	17.95	dirty	88	0.05	0.29	II

Tabarca	36°57'9.09"N 8°46'45.42"E	Tunisia	24.11.21	50	2407	4814	1215	2430	1.98	semi-urban	39.62	extremly dirty	142	0.12	0.69	II
Leedo beach	31°21'30.24"N 27°13'38.33"E	Egypt	16.03.22	30	4533	15095	1465	4878	3.09	urban	61.88	extremly dirty	1341	0.92	5.01	IV
El Graham	31°22'3.05"N 27°13'27.55"E	Egypt	17.03.22	50	3242	6484	2740	5480	1.18	touristic	23.66	extremly dirty	884	0.32	1.84	III
Maamoura beach	31°17'23.73"N 30° 1'29.19"E	Egypt	21.03.22	40	905	2263	1128	2819	0.80	touristic	16.05	dirty	109	0.10	0.65	II
Montaza	31°17'15.60"N 30° 1'7.18"E	Egypt	21.03.22	35	689	1964	525	1496	1.31	urban	26.25	extremly dirty	108	0.21	1.45	III
Montaza 3	31°16'44.04"N 30° 0'27.14"E	Egypt	26.03.22	30	2327	7749	1000	3330	2.33	urban	46.54	extremly dirty	673	0.67	4.00	III
Montaza 2	31°16'40.58"N 30° 0'21.65"E	Egypt	26.03.22	50	3556	7112	875	1750	4.06	urban	81.28	extremly dirty	941	1.08	6.06	IV
Montaza 5	31°15'31.55"N 29°58'42.16"E	Egypt	28.03.22	50	1656	3312	680	1360	2.44	urban	48.71	extremly dirty	213	0.31	1.95	III
Montaza 4	31°15'24.91"N 29°58'40.70"E	Egypt	28.03.22	20	4854	24270	980	4900	4.95	urban	99.06	extremly dirty	1403	1.43	7.77	IV
Medjerda rivermouth	37° 0'40.60"N 10°11'28.84"E	Tunisia	14.06.22	100	436	436	840	840	0.52	semi-rural	10.38	dirty	127	0.15	1.15	III
Cap Angela	37°20'1.79"N 9°45'50.97"E	Tunisia	15.06.22	50	555	1110	2490	4980	0.22	semi-rural	4.46	clean	93	0.04	0.27	II
Bizerte 1	37°16'54.90"N 9°52'53.41"E	Tunisia	16.06.22	20	2255	11275	1700	8500	1.33	urban	26.53	extremly dirty	134	0.08	0.47	II
Hammamet	36°24'7.53"N 10°35'16.97"E	Tunisia	17.06.22	100	1352	1352	1370	1370	0.99	semi-urban	19.74	dirty	195	0.14	0.91	II
Alhambra A	36°21'7.17"N 10°31'42.86"E	Tunisia	19.06.22	50	1454	2908	2505	5010	0.58	semi-urban	11.61	dirty	184	0.07	0.46	II
Hammam Sousse 1	35°51'53.23"N 10°36'51.47"E	Tunisia	21.06.22	50	1019	2038	775	1550	1.31	urban	26.30	extremly dirty	180	0.23	1.54	III
Monastir Skanes	35°45'54.93"N 10°44'2.77"E	Tunisia	22.06.22	30	4610	15351	1440	4795	3.20	touristic	64.03	extremly dirty	422	0.29	1.60	III
Tangier Playa beach	35°46'39.08"N 5°47'39.52"W	Morocco	10.01.23	40	1376	3440	4050	10125	0.34	urban	6.80	moderate	267	0.07	0.42	II
Malabata beach	35°46'44.57"N 5°46'30.93"W	Morocco	11.01.23	100	3758	3758	2200	2200	1.71	urban	34.16	extremly dirty	613	0.28	1.56	III
Oued Alian	35°49'40.32"N 5°38'56.88"W	Morocco	12.01.23	50	883	1766	2126	4252	0.42	semi-rural	8.31	moderate	104	0.05	0.33	II
Oued El Marsa	35°54'17.38"N 5°26'52.06"W	Morocco	13.01.23	60	1078	1800	2246	3751	0.48	semi-rural	9.60	moderate	198	0.09	0.58	II
Fnideq	35°49'5.71"N 5°21'7.31"W	Morocco	14.01.23	60	1039	1735	2580	4309	0.40	semi-urban	8.05	moderate	173	0.07	0.44	II

M Diq	35°41'18.22"N 5°19'26.28"W	Morocco	16.01.23	40	985	2463	1759	4398	0.56	urban	11.20	dirty	183	0.10	0.70	II
Restinga plage	35°45'32.77"N 5°20'40.42"W	Morocco	16.01.23	30	909	3027	1038	3457	0.88	touristic	17.51	dirty	227	0.22	1.48	III
Cabo Negro	35°39'47.90"N 5°17'2.22"W	Morocco	16.01.23	30	1333	4439	1497	4985	0.89	touristic	17.81	dirty	225	0.15	0.96	II
Oued Laou	35°27'15.07"N 5° 5'35.12"W	Morocco	18.01.23	70	3259	4660	3751	5363	0.87	semi-urban	17.38	dirty	997	0.27	1.51	III
Kaa Asreas	35°24'29.95"N 5° 3'48.11"W	Morocco	19.01.23	30	681	2268	2319	7721	0.29	semi-rural	5.87	moderate	109	0.05	0.33	II
Stehat	35°20'44.98"N 4°57'11.11"W	Morocco	19.01.23	50	652	1304	1508	3016	0.43	semi-rural	8.65	moderate	209	0.14	0.98	II
Sabadia Beach	35°14'58.94"N 3°57'10.48"W	Morocco	21.01.23	70	1717	2455	572	817	3.00	urban	60.09	extremly dirty	314	0.55	3.40	III
Isly	35°13'9.81"N 3°54'46.02"W	Morocco	22.01.23	30	1669	5558	986	3283	1.69	semi-rural	33.85	extremly dirty	369	0.37	2.32	III
Quemado	35°14'36.20"N 3°55'35.12"W	Morocco	22.01.23	30	909	3027	717	2388	1.27	urban	25.36	extremly dirty	447	0.62	4.21	IV

Table 2a Sand Rake data with beach name, coordinates, country, survey date, the area surveyed in m², the litter pollution per m², the total amount of litter, and the beach type.

**Name**	**Coordinates**	**Country**	**Date of survey**	**Area in m²**	**Litter per m²**	**Total litter**	**Beach type**
Hammamet 1	36°24'8.44"N 10°35'18.65"E	Tunisia	14.11.2021	26	8.15	212	urban
Hammamet 2	36°24'7.08"N 10°35'14.70"E	Tunisia	14.11.2021	30	2.47	74	urban
Alhambra A1	36°21'7.95"N 10°31'43.18"E	Tunisia	16.11.2021	42	6.07	255	semi-urban
Alhambra A2	36°21'6.65"N 10°31'42.10"E	Tunisia	16.11.2021	40	5.23	209	semi-urban
Alhambra B1	36°21'19.02"N 10°31'51.67"E	Tunisia	16.11.2021	25	4.92	123	touristic
Alhambra B2	36°21'20.31"N 10°31'52.48"E	Tunisia	16.11.2021	27	2.37	64	touristic
Hammam Sousse 1	35°51'59.08"N 10°36'45.53"E	Tunisia	17.11.2021	33	37.45	1236	urban
Hammam Sousse 2	35°51'57.75"N 10°36'46.51"E	Tunisia	17.11.2021	30	16.27	488	urban
Sousse 1	35°49'55.39"N 10°38'31.36"E	Tunisia	18.11.2021	30	55.50	1665	urban
Monastir Qaraiya	35°46'26.32"N 10°50'8.22"E	Tunisia	19.11.2021	25	18.72	468	urban
Bizerte	37°16'58.38"N 9°52'49.53"E	Tunisia	22.11.2021	45	2.67	120	urban

Tabarka 1	36°57'9.60"N 8°46'46.34"E	Tunisia	24.11.2021	30	5.83	175	semi-urban
Tabarka 2	36°57'9.50"N 8°46'44.04"E	Tunisia	24.11.2021	30	3.43	103	semi-urban
Maamoura 1	31°17'23.13"N 30° 1'29.77"E	Egypt	21.03.2022	47.5	9.03	429	touristic
Maamoura 2	31°17'23.50"N 30° 1'28.39"E	Egypt	21.03.2022	50	5.10	255	touristic
Montaza	31°17'16.04"N 30° 1'6.70"E	Egypt	21.03.2022	32.5	2.34	76	touristic
Montaza 1	31°16'39.40"N 30° 0'21.20"E	Egypt	26.03.2022	27	19.93	538	urban
Montaza 2	31°16'41.17"N 30° 0'22.49"E	Egypt	26.03.2022	25.5	10.90	278	urban
Montaza 3	31°16'44.41"N 30° 0'28.76"E	Egypt	26.03.2022	35	29.17	1021	urban
Stanley Bridge 1	31°14'4.10"N 29°56'59.81"E	Egypt	28.03.2022	33	9.03	298	urban
Stanley Bridge 2	31°14'5.95"N 29°57'0.37"E	Egypt	28.03.2022	30	22.87	686	urban
Montaza 5	31°15'31.84"N 29°58'40.98"E	Egypt	28.03.2022	25	23.04	576	urban
Montaza 4	31°15'24.93"N 29°58'41.23"E	Egypt	28.03.2022	40	47.43	1897	urban
Medjerda rivermouth	37° 0'40.64"N 10°11'29.52"E	Tunisia	14.06.2022	26.25	1.60	42	semi-rural
Bizerte	37°16'53.93"N 9°52'52.51"E	Tunisia	16.06.2022	32.5	7.94	258	urban
Hammamet	36°24'9.53"N 10°35'22.50"E	Tunisia	17.06.2022	28	1.61	45	urban
Alhmabra A	36°21'6.65"N 10°31'43.70"E	Tunisia	19.06.2022	25	1.04	26	semi-urban
Hammam Sousse	35°51'53.34"N 10°36'51.78"E	Tunisia	21.06.2022	30	2.53	76	urban
Monastir	35°45'54.98"N 10°44'2.77"E	Tunisia	22.06.2022	25	17.28	432	touristic
Oued Alian	35°49'42.10"N 5°38'49.80"W	Morocco	12.01.2023	27	0.26	7	semi-rural
Oued Alian 2	35°49'38.3"N 5°39'05.9"W	Morocco	12.01.2023	25	0.76	19	semi-rural
Fnideq 1	35°49'18.5"N 5°21'07.2"W	Morocco	14.01.2023	25	2.16	54	semi-urban
Fnideq 2	35°49'14.1"N 5°21'06.8"W	Morocco	14.01.2023	25	2.44	61	semi-urban
Fnideq 3	35°49'10.3"N 5°21'06.9"W	Morocco	14.01.2023	25	0.64	16	semi-urban

Fnideq 4	35°49'05.8"N 5°21'06.3"W	Morocco	14.01.2023	25	1.80	45	semi-urban
Restinga plage 1	35°45'37.2"N 5°20'40.4"W	Morocco	16.01.2023	28	3.21	90	touristic
Restinga plage 2	35°45'33.1"N 5°20'39.9"W	Morocco	16.01.2023	28	1.29	36	touristic
Cabo Negro 1	35°39'52.4"N 5°17'03.2"W	Morocco	16.01.2023	34	3.09	105	touristic
Cabo Negro 2	35°39'47.6"N 5°17'02.8"W	Morocco	16.01.2023	30	4.53	136	touristic
M'Diq 1	35°41'25.7"N 5°19'31.3"W	Morocco	16.01.2023	45	2.09	94	urban
M'Diq 2	35°41'22.5"N 5°19'29.0"W	Morocco	16.01.2023	45	1.98	89	urban
